# Decreased glutamate transport in acivicin resistant *Leishmania tarentolae*

**DOI:** 10.1371/journal.pntd.0010046

**Published:** 2021-12-16

**Authors:** Gaétan Roy, Arijit Bhattacharya, Philippe Leprohon, Marc Ouellette

**Affiliations:** Centre de Recherche en Infectiologie du Centre de Recherche du CHU de Québec and Département de Microbiologie, Infectiologie et Immunologie, Faculté de Médecine, Université Laval, Quebec City, Québec, Canada; University of Liverpool, UNITED KINGDOM

## Abstract

Studies of drug resistance in the protozoan parasites of the genus *Leishmania* have been helpful in revealing biochemical pathways as potential drug targets. The chlorinated glutamine analogue acivicin has shown good activity against *Leishmania* cells and was shown to target several enzymes containing amidotransferase domains. We selected a *Leishmania tarentolae* clone for acivicin resistance. The genome of this resistant strain was sequenced and the gene coding for the amidotransferase domain-containing GMP synthase was found to be amplified. Episomal expression of this gene in wild-type *L*. *tarentolae* revealed a modest role in acivicin resistance. The most prominent defect observed in the resistant mutant was reduced uptake of glutamate, and through competition experiments we determined that glutamate and acivicin, but not glutamine, share the same transporter. Several amino acid transporters (AATs) were either deleted or mutated in the resistant cells. Some contributed to the acivicin resistance phenotype although none corresponded to the main glutamate transporter. Through sequence analysis one AAT on chromosome 22 corresponded to the main glutamate transporter. Episomal expression of the gene coding for this transporter in the resistant mutant restored glutamate transport and acivicin susceptibility. Its genetic knockout led to reduced glutamate transport and acivicin resistance. We propose that acivicin binds covalently to this transporter and as such leads to decreased transport of glutamate and acivicin thus leading to acivicin resistance.

## Author summary

Studies of drug resistance in the protozoan parasites of the genus *Leishmania* have been helpful in revealing biochemical pathways as potential drug targets. Here we report on the characterization at the genomics and metabolomics levels of a *L*. *tarentolae* strain made resistant to acivicin, an analogue of glutamine with activity against this parasite. We found that resistance to acivicin is accompanied by a reduced uptake and intracellular levels of glutamate and that both are expected to share the same transporter. Through gene overexpression and disruption studies we identified the main amino acid transporter responsible for glutamate uptake.

## Introduction

The protozoan parasites of the genus *Leishmania* are distributed worldwide and are responsible for a number of clinical manifestations. A handful number of drugs are available which are unfortunately associated with toxicity, difficulty in administration, cost or drug resistance. There is a need for novel drugs, and there has been many studies investigating different metabolic pathways in *Leishmania* that could eventually be exploited for drug development (reviewed in [1–4]).

While *Leishmania* species are purine auxotroph (reviewed in [5]), they can synthesize *de novo* and salvage pyrimidines [6]. Recent studies of *Leishmania* cells selected for resistance to the pyrimidine analogue 5-fluorouracil revealed a number of salvage enzymes and the capacity of *Leishmania* to transport uracil [7]. In *T*. *brucei*, pyrimidine starvation is lethal but can be rescued by salvage, suggesting that it may not be an essential function for *T*. *brucei* [8]. Recent work, however, has shown that one of the salvage enzymes, thymidine kinase, is essential for parasite viability [9,10] thus suggesting that pyrimidine metabolism may finally indeed be a valid pathway for drug development. The use of drugs has been helpful for understanding biochemical pathways and for example the studies of resistance to the model drugs methotrexate and sinefungin were helpful in our understanding of one-carbon metabolism in *Leishmania* [11–13].

In this study we selected *L*. *tarentolae* for resistance to acivicin. Acivicin is a chlorinated glutamine analogue, first developed as an anti-cancer drug but shown to be highly active against promastigotes and intracellular *L*. *donovani* [14]. Acivicin was shown to target carbamyl phosphate synthase (CPS), the first enzyme of the *de novo* pyrimidine biosynthetic pathway [14]. Protozoan parasites have a unique bifunctional enzyme with a N-terminal glutamine amidotransferase domain fused to the C-terminal CPS domain and the activity of acivicin is due to the inhibition of the glutamine amidotransferase domain [15]. Acivicin was shown to be active against other enzymes containing an amidotransferase domain such as the pyrimidine metabolic enzyme CTP synthase [16] or the purine metabolic enzyme GMP synthase (GMPS) [17]. Acivicin has been shown to be active against *T*. *brucei* by targeting its CTP synthase [16].

We describe here the characterization of *L*. *tarentolae* selected for acivicin resistance by a combination of genomics and metabolomics approaches which revealed a link between acivicin and glutamate accumulation.

## Methods

### Cells lines, culture conditions

*L*. *tarentolae* TarII [18] was grown as promastigotes at 25°C in SDM-79 medium supplemented with 10% heat inactivated fetal bovine serum and 10 μg/mL hemin pH 7.0. The TarII strain was selected for acivicin (Sigma-Aldrich) resistance using a stepwise selection up to 150 μM resulting in the mutant *L*. *tarentolae* A150.1. *L*. *tarentolae* A150.1 was grown without acivicin for 5, 10, and 20 passages to obtain partial revertants.

### DNA manipulation

The genes of interest were amplified from genomic DNA with Phusion Taq polymerase (NEB). After sequencing, to ascertain their correct sequence, the fragments were cloned in the XbaI- HindIII sites of the *Leishmania* expression vectors pSPαHYGα or pSPαNEOα [19]. Gene transfection was carried out by electroporation and transfectants were selected with 1 mM of hygromycin or 0.1 mM of G418. Gene knockout work was performed essentially as described [20] by the integration of *NEO* and *HYG* inactivation cassettes facilitated by the CRISPR-Cas9 system. Southern blot of PFGE and total DNA gels were done by hybridization with [α-^32^P] dCTP-labeled DNA probes according to standard protocols.

### Pulse-field gel electrophoresis (PFGE)

Intact chromosomes were prepared from exponential phase culture of *L*. *tarentolae* and lysed in 1% low melting agarose plugs. The parasites were lysed in MLM buffer (0.5M EDTA pH 9.5, 1% SDS and 350 mg/mL proteinase K) overnight at 50°C. The chromosome were separated in TBE 0.5× (from TBE 10×: 1M Tris, 1M Boric Acid, 0.02 M EDTA) by Pulsed-Field Gel Electrophoresis (PFGE) using a Bio-Rad CHEF-DR III apparatus at 5 V/cm and 120° separation angle as described previously [21].

### Whole genome sequencing and analysis

Paired-ends sequencing libraries were prepared from *L*. *tarentolae* genomic DNA with the Nextera DNA sample prep kit and sequenced on an Illumina HiSeq platform with 101-nucleotides paired-ends reads. Sequence reads were aligned to the *L*. *tarentolae* Parrot TarII PacBio genome assembly [22] using bwa-mem [23]. Read duplicates were marked using Picard and GATK was applied for single nucleotide variants and small insertions or deletions discovery [24]. Copy numbers variations were derived from read depth coverage as described earlier [25].

### Cellular transport assays

Transport assay were performed as previously described [26] with minor modifications. Briefly, for each time point 5 × 10^7^ parasites in exponential growth-phase were washed 3 times with phosphate-buffered saline (PBS) and pelleted. The cell pellet was resuspended in 100 uL of PBS, mixed with an equal volume of different dilutions of cold glutamate or glutamine traced with Tritium-labeled L-[3,4^3^H]-glutamic acid (PerkinElmer) or L-[3,4^3^H(N)]-glutamine (Perkin Elmer) in PBS at room temperature and layered over 100 uL of dibutylphthalate (Sigma) in a 1.5 mL tube. Transport assays were stopped at various time points by spinning the cells through the inert dibutylphthalate layer for 20 seconds at maximum speed in a microcentrifuge. Unincorporated glutamate or glutamine was removed by aspiration and cells were washed twice with 1 mL of cold PBS. Competition studies were carried out using 0.1 mM of cold glutamate traced with 1.0 μCi/assay of L-[3,4-^3^H]- glutamate and competed with 1mM glutamate or glutamine or acivicin. Glutamate and glutamine uptake was counted in a liquid scintillation counter (Beckman) and normalized to cell number. The background transport value was removed by subtracting the accumulation value obtained on ice. Incorporated glutamate or glutamine was calculated as follows: Glu(or Gln)_i_ = cpm_i_ × [Glu (or Gln)] × ν × cpm_*st*_^-1^ × *t*^-1^ where Glu_i_ (or Gln_i_) is the incorporated glutamate or glutamine, [Glu] or [Gln] is the nanomolar concentration of radiolabeled glutamate or glutamine, ν is the volume of radiolabeled glutamate or glutamine (100 uL in all cases), cpm_*st*_ is the total cpm measured for the added radiolabeled glutamate or glutamine and *t* is the time of incubation measured in minutes.

### Glutamate and glutamine measurements in *Leishmania* by LC-MS

The amino acids glutamate and glutamine were quantitated essentially as described [27] by liquid chromatography (LC) on an Acquity UPLC I-Class binary pump system and mass spectrometry (MS) on a Q-TOF Synapt G2-*Si* system with an electrospray ionization source in positive ionization mode. The relative concentrations of intracellular glutamine and glutamate in metabolite extracts were determined against calibration curves.

### Statistical analysis

Growth curves were performed in biological triplicates (unless specified otherwise), each performed with technical triplicates. For statistical analysis a two-tailed unpaired t-test with GraphPad Prism software was performed unless mentioned otherwise.

## Results

### *Leishmania tarentolae* acivicin resistant mutant and gene copy number variation

**Fig 1:**
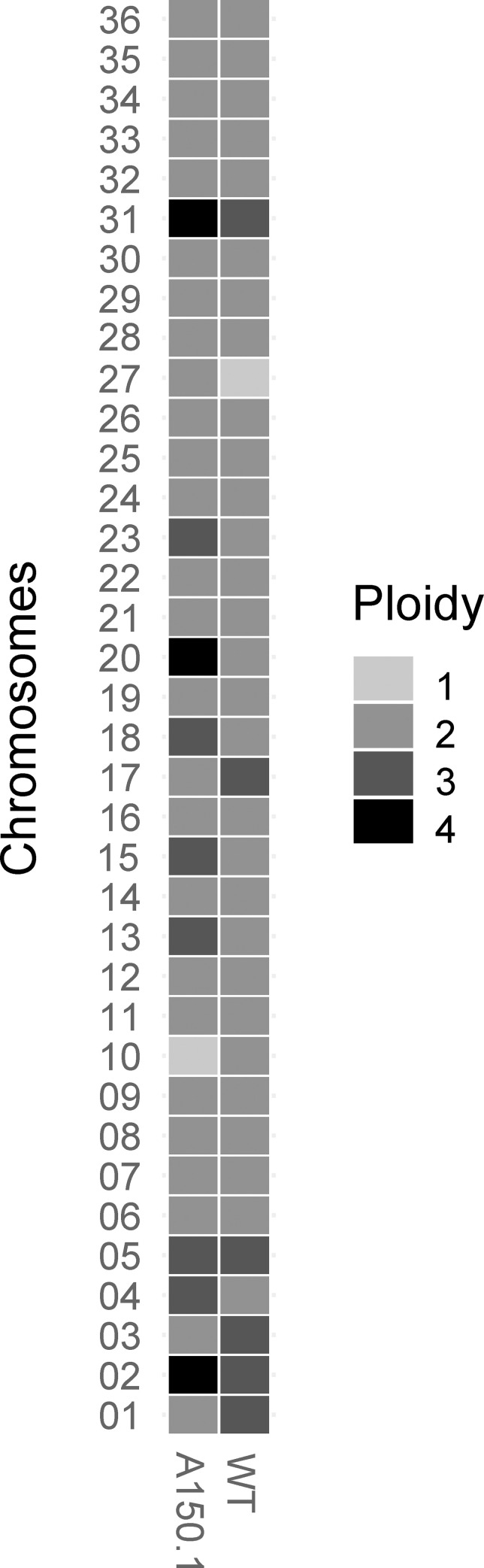
Polyploidy of chromosomes in *L. tarentolae* wild-type and A150.1 cells. The number of alleles per chromosome were inferred from the sequencing data.

*L*. *tarentolae* promastigotes were susceptible to acivicin in the sub μM range (Table 1). One *L*. *tarentolae* clone was selected step by step for acivicin resistance up to 150 μM (*L*. *tarentolae* A150.1) and was close to 100-fold more resistant than the wild-type (WT) cell (Table 1). We sequenced the genome of *L*. *tarentolae* A150.1 that was compared to the *L*. *tarentolae* WT genome sequence [28] re-sequenced by long-reads sequencing [22]. A total of 20.7M reads were obtained for the mutant, producing a genome assembly of 31.2 Mb with a coverage depth of ≥30 fold for more than 90% of the nucleotides. Copy number variations (CNVs) were identified by comparing the coverage of uniquely mapped reads between *L*. *tarentolae* A150.1 and the WT line. Several cases of supernumerary chromosomes were observed in *L*. *tarentolae* A150.1 (Fig 1). Most of these had a gain of 1.5 chromosome copies compared to WT parasites, with the exception of chromosome 20 which is tetraploid in *L*. *tarentolae* A150.1 and thus gained 2 copies compared to *L*. *tarentolae* WT. The *L*. *tarentolae* A150.1 mutant also lost one allele for a few chromosomes (Fig 1).

**Table 1:** Susceptibility to acivicin for the L. tarentolae strains used in this study.

Strains	Vector(s)	Genes	EC50 (μM)^a^	Resistance index^b^
L. tarWT	-	-	0.92±0.02	-
L. tarWT	pSPαHYGα	-	0.79±0.02	ns
L. tarWT	pSPαHYGα-Cas9	-	0.81±0.1	ns
L. tarA150.1	-	-	95.97±10.93***^c^	100×^c^
L. tarA150.1	pSPαHYGα	-	74.58±5.02***^d^	95×^d^
L. tarWT_GMPS	pSPαHYGα- LtaPh_2201100	GMP synthase	1.41±0.01*^d^	1.80×^d^
L. tarWT_MFS	pSPαHYGα- LtaPh_3528000	Major facilitator Superfamily	0.98±0.06	ns
L. tarA150.1_0712500	pSPαHYGα- LtaPh_0712500	AAT19 subfamily	78.8 ±6.6	ns
L. tarA150.1_2202300	pSPαHYGα- LtaPh_2202300	AAT22 subfamily	18.2±8.2***^e^	0.20×^e^
L. tarA150.1_3106161	pSPαHYGα- LtaPh_3106161	AAT1 subfamily	27.3±11.3** ^e^	0.35×^e^
L. tarA150.1_3103700	pSPαHYGα- LtaPh_3103700	AAT1 subfamily	84.2±5.1	ns
L. tarA150.1_3646100	pSPαHYGα- LtaPh_3646100	AAT14 subfamily	86.2±12.1	ns
L. tarWT_Δ2202300	pSPαHYGα-Cas9	AAT22 subfamily	1.32±0.07** ^f^	1.60×^f^

a EC50 ± standard deviation; p value ≤ 0.05 (*), p value ≤ 0.01 (**), p value ≤ 0.001 (***)

b The fold resistance to acivicin is indicated only for genes conferring a statistically significant difference compared to the proper control. ns, not significant.

c In comparison to *L. tarentolae* WT; n = 5

d In comparison to *L. tarentolae* WT transfected with pSPαHYGα; n = 5

e In comparison to *L. tarentolae* A150.1 transfected with pSPαHYGα; n = 5

f In comparison to *L. tarentolae* transfected with pSPαHYGα-Cas9; n = 5

**Fig 2:**
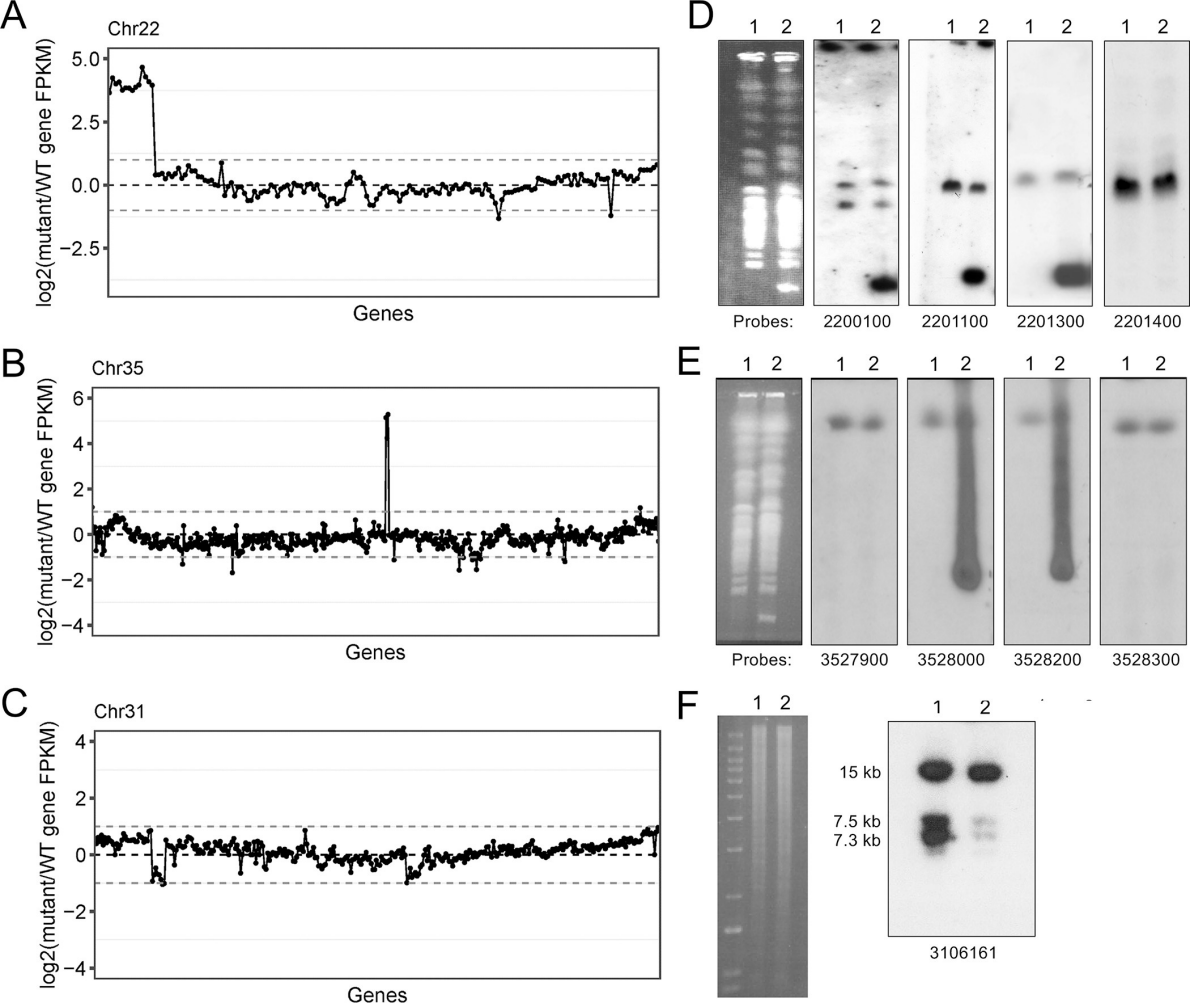
Gene amplification and deletion in *L. tarentolae* A150.1. Log2-transformed *L. tarentolae* A150.1/WT reads ratios revealed amplification of genomic segments on chromosome 22 (A) and 35 (B), as well as gene deletion on chromosome 31 (C). Southern blot hybridizations of chromosomes separated by pulse-field gel electrophoresis using 32P-labelled DNA probes derived from chromosome 22 (D) and 35 (E). Southern blot of genomic DNAs digested with NheI and hybridized to a LtaPh_3106161 probe (F). The bands at 7.3 and 7.5 Kb correspond to LtaPh_3106161 while the band at 15 Kb corresponds to at least one AAT homologue that is not deleted. The ethidium bromide gel on the left shows equal lane loading. For panels D to F, the probes used for hybridization correspond to the complete open reading frame for the genes indicated under the blots. The gene sequence of LtaPh_2200100 in panel D shares a high homology with the gene LtaPh_0613000 which likely explains the two bands observed for both A150.1 and WT. Lane 1, *L. tarentolae* WT; Lane 2, *L. tarentolae* A150.1.

Normalized read depth coverage allowed the identification of genomic loci that were amplified or deleted in *L*. *tarentolae* A150.1. These are characterized by stretches of genes whose normalized read coverage abruptly varies compared to the WT (S1 Fig and S1 Table). Among the most striking observations were a subtelomeric region of ~55 kb on chromosome 22 that was amplified ~16-fold in *L*. *tarentolae* A150.1 based on the normalized read counts (Fig 2A). Amplification of smaller regions on chromosomes 35 (~32 fold) (Fig 2B) and 15 (~4-fold) (S1 Fig) and deletions on chromosome 31 (~2-fold) (Fig 2C) and at the end of chromosome 29 (~2 fold) (S1 Fig) were also observed. The profile of the different chromosomes suggests several other genomics events but these are either of a lesser fold difference or covering a single gene (S1 Fig), the latter of which should be considered with caution.

The change in copy number of specific genomic regions was confirmed by Southern blots. When we migrated the chromosomes of *L*. *tarentolae* cells in a pulse field gel (PFGE) we could readily see a low molecular weight band, smaller than the smallest chromosome, in *L*. *tarentolae* A150.1 which was absent in the WT cells (Fig 2D). The migration profile of this extra band is a signature for linear amplification [29]. It corresponds to the CNV observed for chromosome 22 (Fig 2A), since this amplicon hybridized with a number of probes spanning chromosome 22 (Fig 2D). The hybridization data was consistent with the breakpoint observed with sequence reads along chromosome 22, the linear amplicon extending from the telomeric sequences up to LtaPh_2201300 (Fig 2D and S1 Table).

Our CNV analysis also highlighted a small region amplified on chromosome 35 and hybridization with probes derived from the region indeed confirmed the amplification of a locus containing 3 genes as part of extrachromosomal circles, as suggested by the signature hybridization smear in *L*. *tarentolae* A150.1 (Fig 2E). The deletion of a genomic segment covering 10 genes on chromosome 31 revealed by the sequencing data (Fig 2C) was also supported by a Southern blot of NheI digested DNAs hybridized to a LtaPh_3106161 probe (Fig 2F). The product of this gene is part of a family of amino acid transporters (AATs) and would fall into the AAT1 subfamily according to the phylogenetic analysis and nomenclature of Jackson [30].

### Single nucleotide polymorphisms in *L*. *tarentolae* acivicin resistant A150.1 mutant

Outside CNVs, single nucleotide polymorphisms (SNPs) are also important contributors of drug resistance in *Leishmania* [31]. The genome of *L*. *tarentolae* A150.1 had a total of 2336 SNPs compared to *L*. *tarentolae* WT, of which 416 were in genes and non-synonymous. Of these, 51 were homozygous and 365 were heterozygous. A total of 2519 small insertions/deletions (indels) were also detected, of which 5 were in genes and homozygous and 85 were in genes and heterozygous. The list of homozygous and heterozygous SNPs and indels in coding sequences is provided in S2 Table. The list of SNPs and indels within intergenic regions can be found in S3 Table. Since acivicin is a glutamine analogue we noticed SNPs in three AATs, including the homozygous change in LtaPh_3646100 and LtaPh_3106161, and the heterozygous substitutions in LtaPh_0712500 (S2 Table), belonging respectively to the AAT14, AAT1 and AAT19 subfamilies [30]. Another mutation potentially linked to acivicin resistance (see discussion) consists in the homozygous change in the glucosamine-fructose-6-phosphate aminotransferase LtaPh_0609200 (S2 Table).

### Genes involved in acivicin resistance

We first concentrated on genes with altered CNVs and that could produce resistance to acivicin. The linear episome derived from chromosome 22 contains 14 genes (LtaPh_2200051 to LtaPh_2201300) and one of them, LtaPh_2201100, encodes for the *Leishmania* GMPS orthologue. This is one known target of acivicin and we directly tested its role by cloning it into an expression vector that was transfected in *L*. *tarentolae* WT cells. The *L*. *tarentolae* GMPS transfectant was 1.8 fold more resistant to acivicin than the control cells (Table 1). This was a minor albeit statistically significant contribution to resistance. The identity of the other genes co-amplified with GMPS did not suggest a role for any of them in acivicin resistance. There were only three genes (LtaPh_3528000-LtaPh_3528200) on the circular amplicon derived from chromosome 35 but transfection into *L*. *tarentolae* WT cells did not confer increased resistance to acivicin (Table 1).

**Fig 3:**
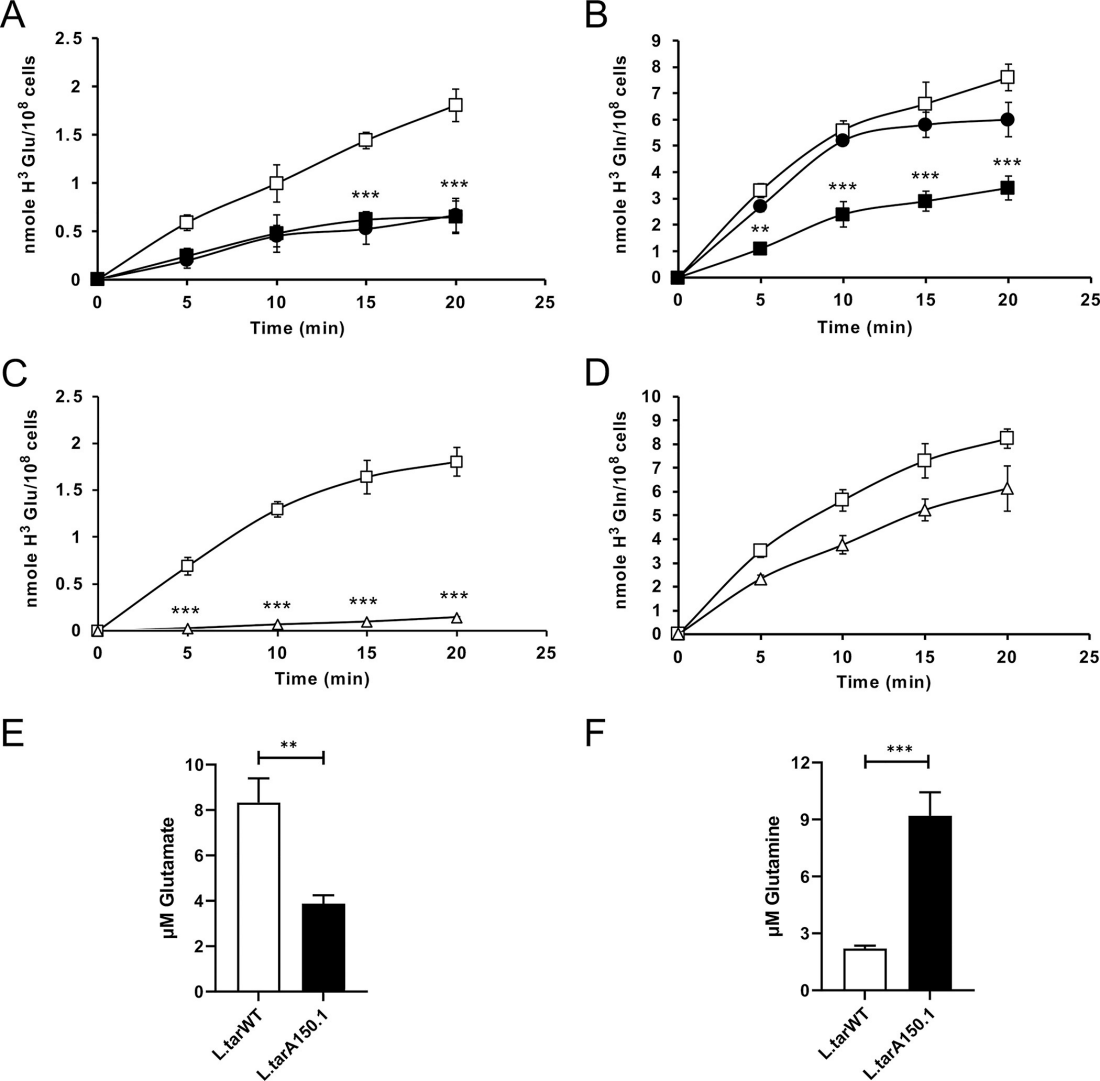
Accumulation of glutamine and glutamate in *L. tarentolae* WT and A150.1. (A) Time-dependent uptake of 0.1mM glutamate traced with L-[3,43H]-glutamate in *L. tarentolae* WT pSPαHYGα alone (open squares) and in the presence of 1mM acivicin (filled circles) or 1mM unlabelled glutamate (filled squares). (B) Time-dependent uptake of 0.1mM glutamine traced with L-[3,43H]-glutamine in *L. tarentolae* WT pSPαHYGα alone (open squares) and in the presence of 1mM acivicin (filled circles) or 1mM unlabelled glutamine (filled squares). (C) Time-dependent uptake of 0.1mM glutamate traced with L-[3,43H]-glutamate in *L. tarentolae* WT (open squares) and A150.1 (open triangles). (D) Time-dependent uptake of 0.1mM glutamine traced with L-[3,43H(N)]-glutamine in *L. tarentolae* WT (open squares) and A150.1 (open triangles). Levels of intracellular glutamate (E) and glutamine (F) in *L. tarentolae* WT and A150.1 as measured by liquid-chromatography coupled to mass spectrometry. ** p<0.01; ***p<0.001.

**Fig 4:**
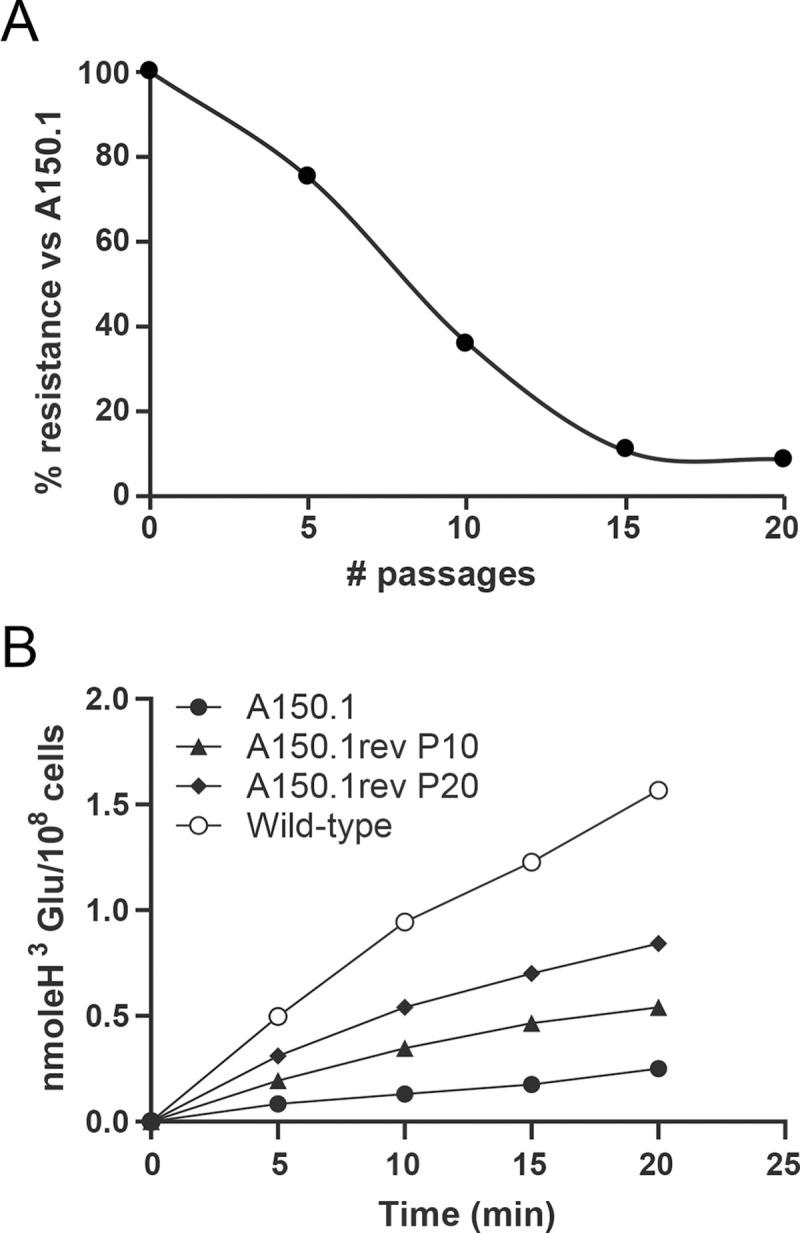
Gradual increase in sensitivity to acivicin and glutamate transport in *L. tarentolae* A150.1 grown in absence of acivicin. The *L. tarentolae* A150.1 mutant was grown for 5, 10 and 20 passages in absence of acivicin. At those passages, the level of acivicin resistance relative to A150.1 (A) and glutamate accumulation (B) were measured. For the latter, we measured the time-dependent uptake of 0.1mM glutamate traced with L-[3,43H]-glutamate.

The sequence data revealed two deletions on chromosome 31 that covered putative AATs (Fig 2C and S1 Table). Chromosome 31 is polyploid (Fig 1) and the deletion signals are coherent with the genes being loss from at least half of chromosome 31 alleles in *L*. *tarentolae* A150.1 (Fig 2C and 2F and S1 Table). A decrease in copy number of putative amino acid transporter genes prompted us to measure the transport of glutamate and glutamine since these are close analogues to acivicin. We first confirmed that *L*. *tarentolae* accumulates both glutamate (Fig 3A) and glutamine (Fig 3B). Different gene products possibly transport these two amino acids since acivicin competed with glutamate (Fig 3A) but not glutamine (Fig 3B) accumulation. Acivicin seems to compete equally well as cold glutamate (Fig 3A). Interestingly, the accumulation of glutamate, but not glutamine, was greatly diminished in *L*. *tarentolae* A150.1 (Fig 3C and 3D). The intracellular levels of glutamate and glutamine were also calculated in *L*. *tarentolae* WT and A150.1 by quantitative metabolomics using liquid-chromatography mass spectrometry (LC-MS) [27]. The level of glutamate was decreased by 2-fold in *L*. *tarentolae* A150.1 (Fig 3E) while the level of glutamine was 5-fold higher in the mutant compared to *L*. *tarentolae* WT (Fig 3F). We also monitored the stability of the resistance phenotype and we found that part of the resistance was lost with the number of passages in the absence of acivicin (Fig 4A). Interestingly this partial loss in resistance was paralleled with an increased capacity of transporting glutamate (Fig 4B).

**Fig 5:**
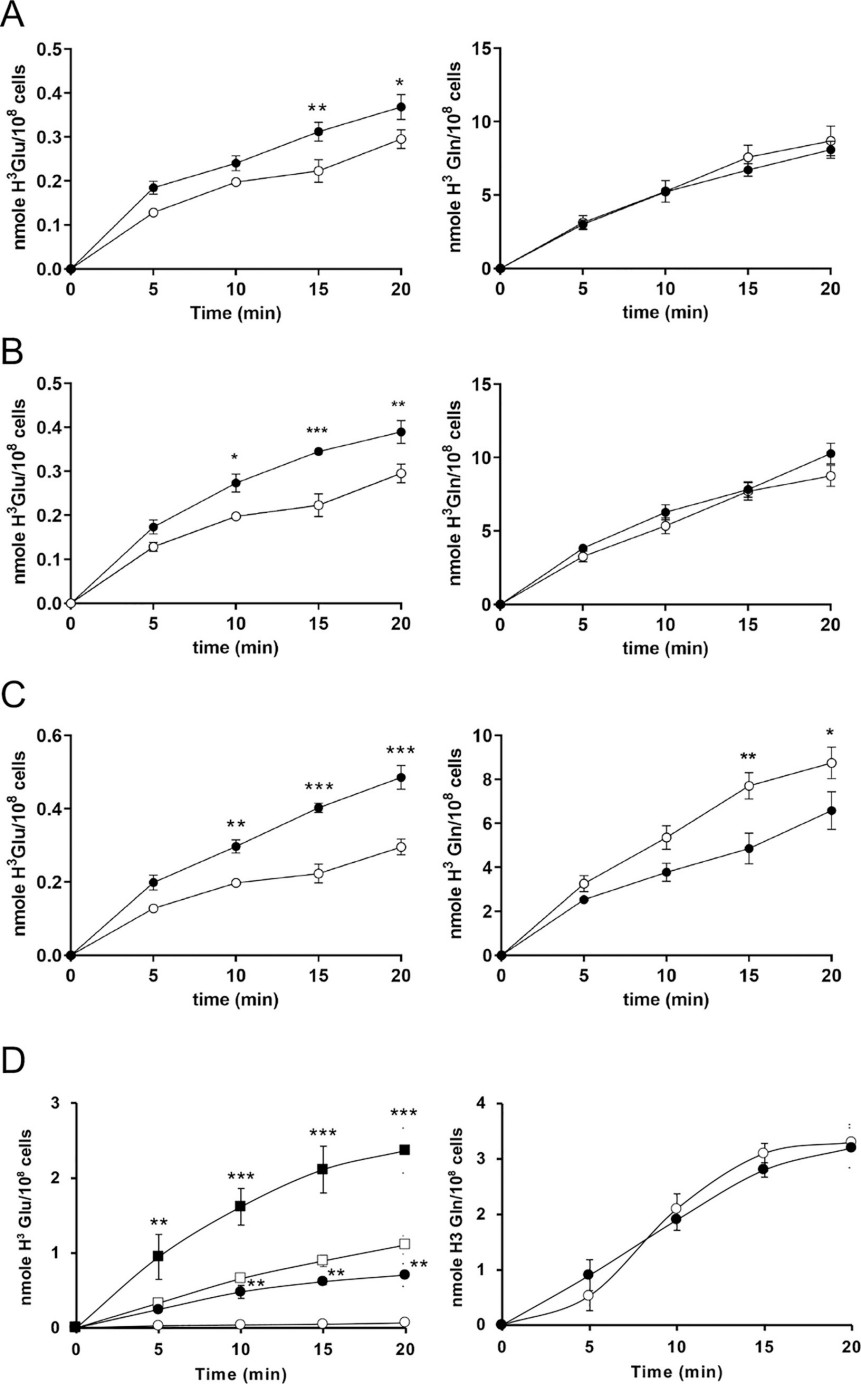
Accumulation of glutamate and glutamine in *L. tarentolae* A150.1 and in its transfectants with amino acid transporter (AAT) genes. Time-dependent uptake of 0.1mM glutamate (left panels) or glutamine (right panels) traced with respectively L-[3,43H]-glutamate or L-[3,43H(N)]-glutamine in the *L. tarentolae* A150.1 mutant transfected with either a control vector (empty circles) or with LtaPh_3106161 (A), LtaPh_07.12500 (B), LtaPh_36.46100 (C), LmjF22.0230 (D) (filled circles). For the latter (D) we also show the accumulation of glutamate in wild-type cells transfected with LmjF22.0230 (filled squares) or with the control vector (empty squares). * p<0.05; ** p<0.01; ***p<0.001. For glutamate in panel D the level of significance indicated is for the comparison between mock-transfected and LmjF.22.0230-transfected cells (empty vs filled symbols) of the same strain (circles or squares). We noticed that the uptake of glutamate and glutamine in panel D were slightly lower in the wild-type control in comparison to what shown in Fig 3A and 3B, but overall glutamate uptake is active in the wild-type and not in the mutant A150.1.

Overexpression of the AAT1 LtaPh_3106161, whose gene copies are partly deleted in A150.1 (Fig 2C and 2F and S1 Table), in *L*. *tarentolae* A150.1 did not alter glutamine accumulation but exhibited a modest but significant increase in glutamate accumulation (Fig 5A). Overexpression of LtaPh_3106161 in A150.1 did revert acivicin resistance by 3-fold (Table 1), supporting the phenotypic consequences of its deletion in A150.1. This re-sensitization is specific since overexpression of the close homologue LtaPh_3103700 did not revert resistance (Table 1). Mutations (SNPs) were found in two additional putative amino acids transporters (LtaPh_0712500 and LtaPh_3646100) (S2 Table). Transfection of LtaPh_0712500 in *L*. *tarentolae* A150.1 did not impact glutamine transport but similarly to the AAT1 LtaPh_3106161 it did increase slightly glutamate accumulation (Fig 5B). It did not however impacted acivicin resistance (Table 1). Transfection of LtaPh_3646100 in A150.1 was intriguingly associated with a small decrease in glutamine accumulation but in this transfectant the accumulation of glutamate was significantly increased (Fig 5C). While the transfection of the WT versions of these three AATs (deleted or mutated in A150.1) in *L*. *tarentolae* A150.1 reverted partly the defect in glutamate transport in A150.1, none of these AATs appear to qualify as the main glutamate transporter.

**Fig 6:**
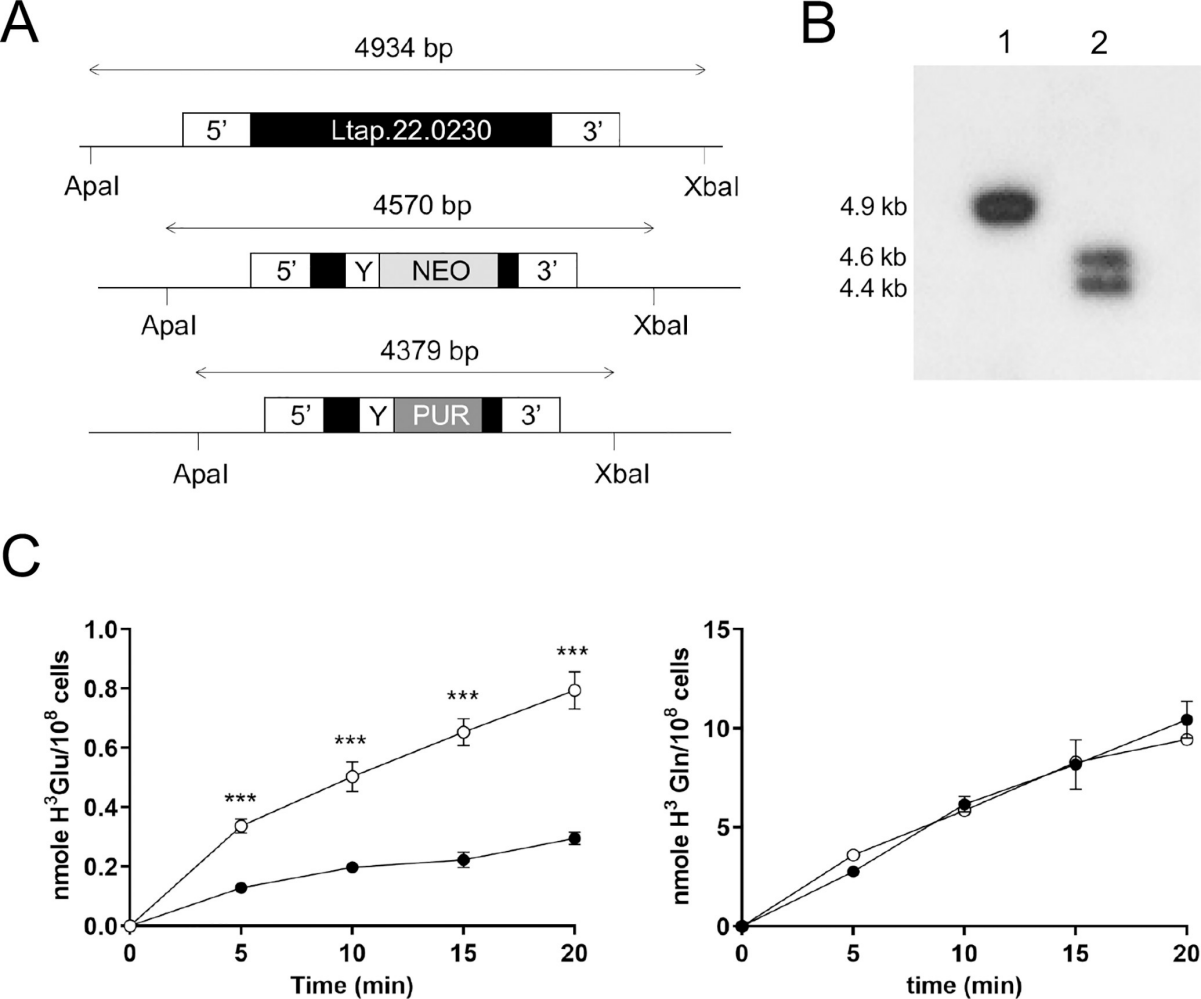
Inactivation of the glutamate transporter LtaPh_2202300. (A) Genomic organization of the LtaPh_2202300 locus. LtaPh_2202300 upstream and downstream fragments used for the integration of the NEO and PUR cassettes are shown. The integration of these cassettes was mediated by a CRIPR-Cas9 strategy [20]. (B) Southern blots of *L. tarentolae* genomic DNA digested with ApaI and XbaI and hybridized to a LtaPh_2202300 5’UTR probe. Lane 1, *L. tarentolae* wild type; lane 2, *L. tarentolae* LtaPh_2202300 null mutant with integrated NEO and PUR cassettes. (C) Time dependence uptake of 0.1mM glutamate traced with L-[3,43H]-glutamate (left) and of 0.1mM glutamine traced with L-[3,43H(N)]-glutamine (right) in the LtaPh_2202300 null mutant (filled circles) or the control strain (empty circles). ***p<0.001.

Members of the solute carrier (SLC) proteins are structurally and functionally diverse and some subclasses were shown to transport amino acids using H(+) as the major driving force. Active transport of glutamate in *Leishmania* appears to be sensitive to extracellular concentration of H(+) but not to Na(+) [32]. When we aligned sequences of *Leishmania* putative amino acid permeases of unknown function we found that LtaPh_2202300 (LmjF22.0230) (AAT22 according to [30]) had 27% identity to SLC38A6 (SNAT6) (S2 Fig), a transporter with properties that could qualify it as a transporter of glutamate and which is conserved among *Leishmania* species (S3 Fig). We studied the *L*. *major* orthologue, as there were gaps with the *L*. *tarentolae* sequence genome draft when this work was started. We thus cloned the *L*. *major* orthologue LmjF22.0230 AAT22 in an expression vector that was transfected into *L*. *tarentolae* WT and A150.1. While episomal expression of LmjF22.0230 did not change the transport properties to glutamine in *L*. *tarentolae* WT (Fig 5D), the transport of glutamate was higher (Fig 5D). Transfection of LmjF22.0230 also restored glutamate transport in *L*. *tarentolae* A150.1 close to wild-type levels (Fig 5D). This regained capacity of transporting glutamate was associated with partial reversion of acivicin resistance (Table 1). In light of this result we reanalysed the sequence of AAT22 in A150.1 but could not detect either SNPs or CNVs, confirming our initial analysis that this gene is unaltered. To further the role of AAT22 in glutamate transport we inactivated LtaPh_2202300 using the CRISPR-Cas9 genome editing technology. Two inactivation cassettes with the NEO and PUR selectable markers were designed (Fig 6A) and transfected along with an episome driving the expression of a guide RNA targeting LtaPh_2202300 in a *L*. *tarentolae* WT cell line expressing the Cas9 nuclease [20]. A null mutant of LtaPh_2202300 was obtained as illustrated by a Southern blot analysis and the absence of an AAT22 WT allele (Fig 6B). The accumulation of glutamate but not glutamine was greatly diminished in that recombinant strain (Fig 6C). This strain was 1.6-fold more resistant to acivicin (Table 1).

## Discussion

Our characterization of a *L*. *tarentolae* mutant selected for acivicin resistance indicates that resistance is complex with many factors contributing modestly to the resistance phenotype. The most prominent defect was a decreased accumulation of glutamate in this mutant. Indeed, acivicin appears to be transported by the glutamate transporter and not by the glutamine transporter. Very little is known about glutamate transport in *Leishmania* and kinetoplastid parasites. It was characterized biochemically in *L*. *amazonensis* [32] and in *T*. *cruzi* [33] but the identity of the transporters remains unknown. One well studied AAT is the *L*. *donovani* AAP24 protein (AAT20 according to [30]) responsible for proline uptake [34] and while it regulates homeostasis of glutamate, the latter is not a substrate. Next generation sequencing is now a useful technique for analysing *Leishmania* drug resistant mutants but in the case of *L*. *tar* A150.1 genomics could not explain the decreased glutamate, and hence most likely acivicin, accumulation. While the genome sequence of the A150.1 mutant revealed many candidates that could explain this uptake defect (e.g. three AAT genes were either deleted or mutated) none appears to play a major role in glutamate accumulation. To gain insight into the main transporter of glutamate (and acivicin) we then resorted to phylogenetic analyses of AAT orthologs. This led to the gene LtaPh_2202300 (AAT22), which is neither mutated nor changed in copy number in A150.1, as the main glutamate transporter. Indeed, transfecting AAT22 in A150.1 restored glutamate transport while glutamate transport was diminished significantly in a *L*. *tarentolae* LtaPh_2202300 knockout cell line. Yet, we still observed glutamate transport in the AAT22 null mutant and thus there are other routes by which glutamate can enter into the cell. The 3 AAT genes found mutated or deleted in A150.1 were associated with low glutamate transport (Fig 4A–4C) and this likely explains why the transport of glutamate is lower in the mutant A150.1 in comparison to the AAT22 knockout.

How can we explain that AAT22 is not mutated in the acivicin resistant mutant A150.1? Acivicin is known to bind covalently to many of its enzymatic targets [35–37] and it was even advocated as a model molecule for the design of covalent enzymatic inhibitors [38]. Thus acivicin may covalently binds the transporter hence reducing its activity. Our observation that glutamate uptake and intracellular levels are diminished in cells resistant to acivicin (Fig 3) is consistent with this hypothesis. Cells can live without the main glutamate transporter, the covalent binding of acivicin to the transporter is likely to be sufficient to reduce its activity, and therefore there is no need for mutation or deletion of this gene. It is possible that acivicin does not bind as strongly to other AAT transporters and thus they need to be mutated or deleted to reduce the uptake of acivicin and by ricochet of glutamate. The resistance reversion work (Fig 4) is providing further support for the covalent binding hypothesis of a drug to a transport protein as a novel mechanism of drug resistance in *Leishmania*. Indeed, with increased passages in the absence of acivicin we observed a decreased resistance to acivicin which was correlated with increased accumulation of glutamate (and most likely acivicin) (Fig 4). Since the main glutamate transporter is neither deleted nor mutated in this mutant it suggests that with gradual passages more of the AAT transporter is acivicin free and its function is gradually restored. Transport is not to wild-type levels because other transporters are altered. Modeling and docking experiments with acivicin and glutamate could be done to support our hypothesis but the lack of a protein structure sufficiently similar to AAT22 is preventing for now such analysis.

In addition to the defect in glutamate transport we found that one potential target, GMPS, is amplified in A150.1. Its transfection only show low level resistance, however. While another locus encoded on chromosome 35 was also amplified as part of an extrachromosomal circle, we could not find a role in acivicin resistance for any of the three genes tested. Usually gene amplification is linked to a phenotype in drug resistant *Leishmania* cells [39] and one gene part of the chromosome 35 locus may need another partner for producing resistance. One other mutation potentially linked to acivicin resistance include the homozygous change in the glucosamine-fructose-6-phosphate aminotransferase LtaPh_0609200 (S2 Table) which if non-functional would divert glutamine away from the synthesis of glutamate and D-glucosamine-6-P, leading to the accumulation of glutamine and to a shortage of glutamate. Our measure of glutamate and glutamine levels by LC-MS (Fig 3E and 3F) are consistent with such hypothesis. We were not able to reconstruct the level of resistance found in the mutant A150.1. Possibly a number of mutations are acting synergistically. This would need to be tested, and the sequencing of the genome of cells at intermediate steps of acivicin selection could help in finding the right order of combination of genes to be transfected to reconstruct resistance.

Our study of acivicin resistance in *L*. *tarentolae* has highlighted the complexity of mutations involved in resistance to a single drug and has allowed the discovery that acivicin uptake is linked to glutamate uptake which led to the discovery of the *Leishmania* glutamate transporter. We propose that acivicin binds covalently to the glutamate transporter and this binding contribute to the reduction in glutamate (and acivicin) uptake and to decreased susceptibility to acivicin.

## Supporting information

S1 Fig Chromosomal amplification and deletion in *L*. *tarentolae* A150.1. Log_2_-transformed *L*. *tarentolae* A150.1/WT gene reads ratios normalized for gene length and total read counts for the 36 chromosomes. Genes along the chromosome are shown as dots on the X axis while the log_2_-transformed *L*. *tarentolae* A150.1/WT reads ratios are indicated by the y axis.
(PDF)

S2 Fig Alignment of SNAT6 homologs. Alignment of the *Homo sapiens* solute carrier family 38 member 6 (SLC38A6), *Trypanosoma cruzi* strain CL Brener gene TcCLB.507811.100, *Rattus norvegicus* solute carrier family 38 member 6 (Slc38a6) and *L*. *major* strain Friedlin gene LmjF22.0230. Blue represents residues A, C, F, I, M, V and W; Dark Blue represents residues H and Y; Pink represents residues E and D; Green represents residues N, Q, S and T; Yellow represents residue P; Orange represents residue G; Coral represents residues K and R; ‘*’ indicates position which have a single fully conserved residue; ‘:’indicates a strong group of conserved amino acids; ‘.’ indicates a weaker group of conserved amino acids.
(TIF)

S3 Fig Phylogeny of AAT22 in *Leishmania ssp*. The evolutionary history was inferred using the Neighbor-Joining method. The percentage of replicate trees in which the associated taxa clustered together in the bootstrap test (500 replicates) are shown next to the branches. The tree is drawn to scale, with branch lengths in the same units as those of the evolutionary distances used to infer the phylogenetic tree. The evolutionary distances were computed using the Poisson correction method and are in the units of the number of amino acid substitutions per site. All ambiguous positions were removed for each sequence pair (pairwise deletion option). Orthologs of AAT22 in *Leptomonas seymouri*, *Crithidia fasciculata* and *Trypanosoma cruzi* were used as outgroup.
(TIF)

S1 Table Genes with an increased (spreadsheet A) or decreased (spreadsheet B) copy number in *L*. *tarentolae* A150.1 as measured by read counts ratios in comparison to *L*. *tarentolae* WT.
(XLSX)

S2 Table List of SNVs detected in the coding regions of *L*. *tarentolae* A150.1. (XLSX)

S3 Table List of SNVs detected in the intergenic regions of *L*. *tarentolae* A150.1. (XLSX)
