# Supplementary material for: Decreased glutamate transport in acivicin resistant Leishmania tarentolae
Source: PLoS Negl Trop Dis. 2021 Dec 16;15(12):e0010046. doi: 10.1371/journal.pntd.0010046 (PMC8718007; doi:10.1371/journal.pntd.0010046)
Supplement: S1 Fig — Log2-transformed L. tarentolae A150.1/WT gene reads ratios normalized for gene length and total read counts for the 36 chromosomes. Genes along the chromosome are shown as dots on the X axis while the log2-transformed L. tarentolae A150.1/WT reads ratios are indicated by the y axis. (PDF) [file pntd.0010046.s001.pdf]

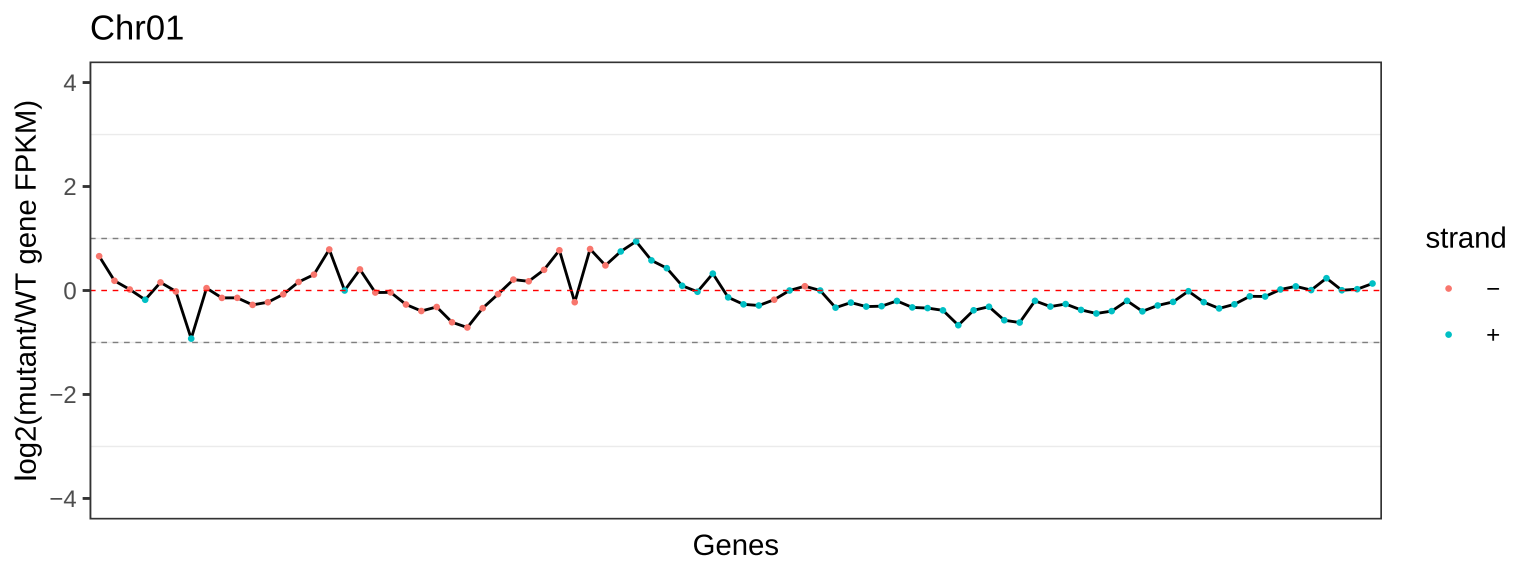

Chr01
4
2
0
−2
−4
log2(mutant/WT gene FPKM)
Genes
strand
−
+

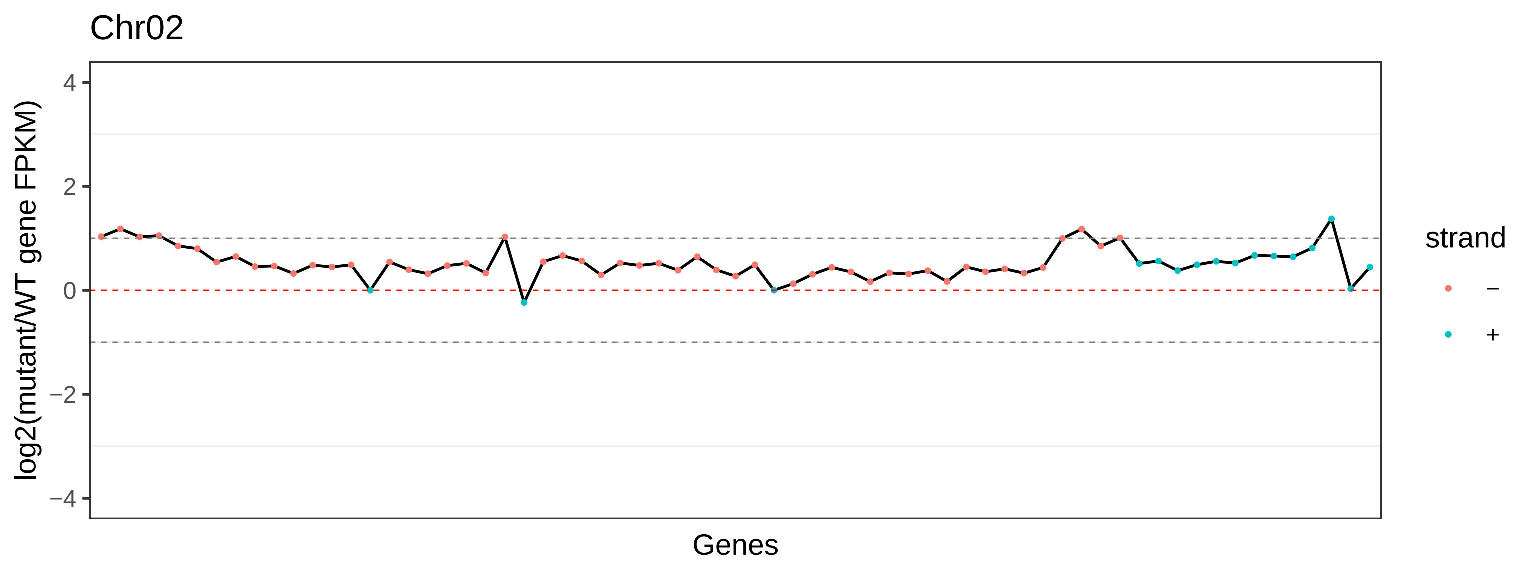

Chr02
log2(mutant/WT gene FPKM)
4
2
0
−2
−4
Genes
strand
−
+

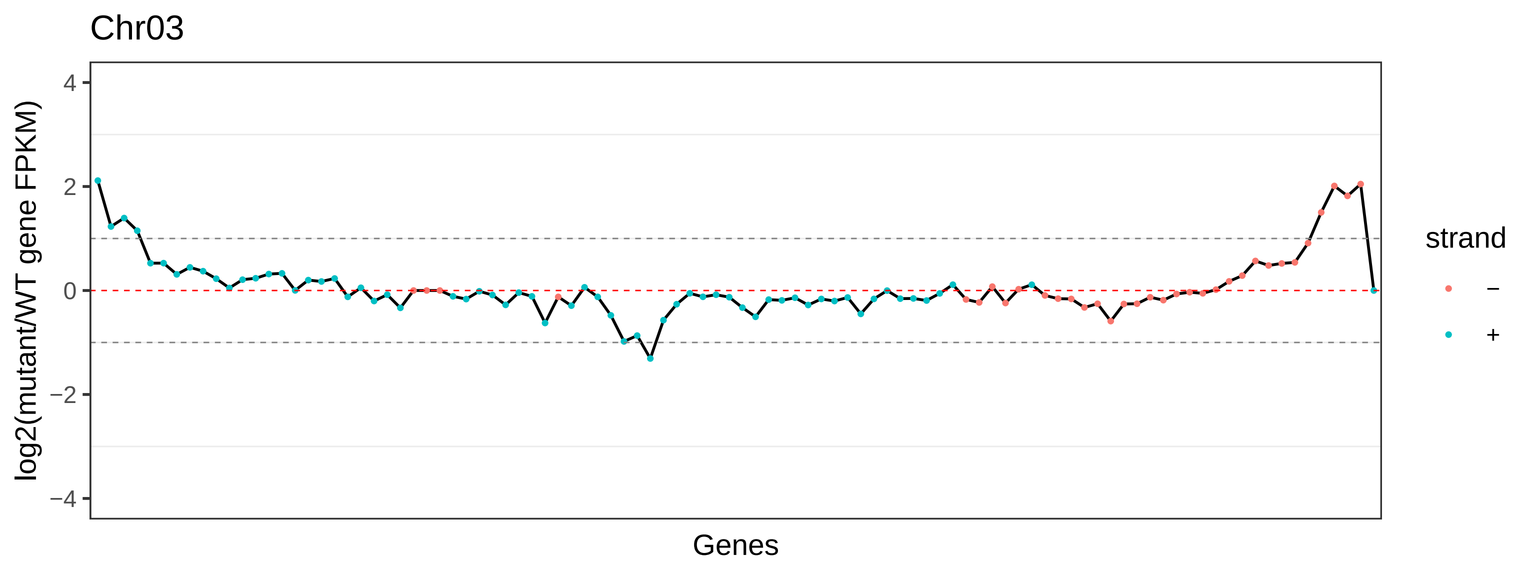

Chr03
4
2
0
−2
−4
log2(mutant/WT gene FPKM)
Genes
strand
−
+

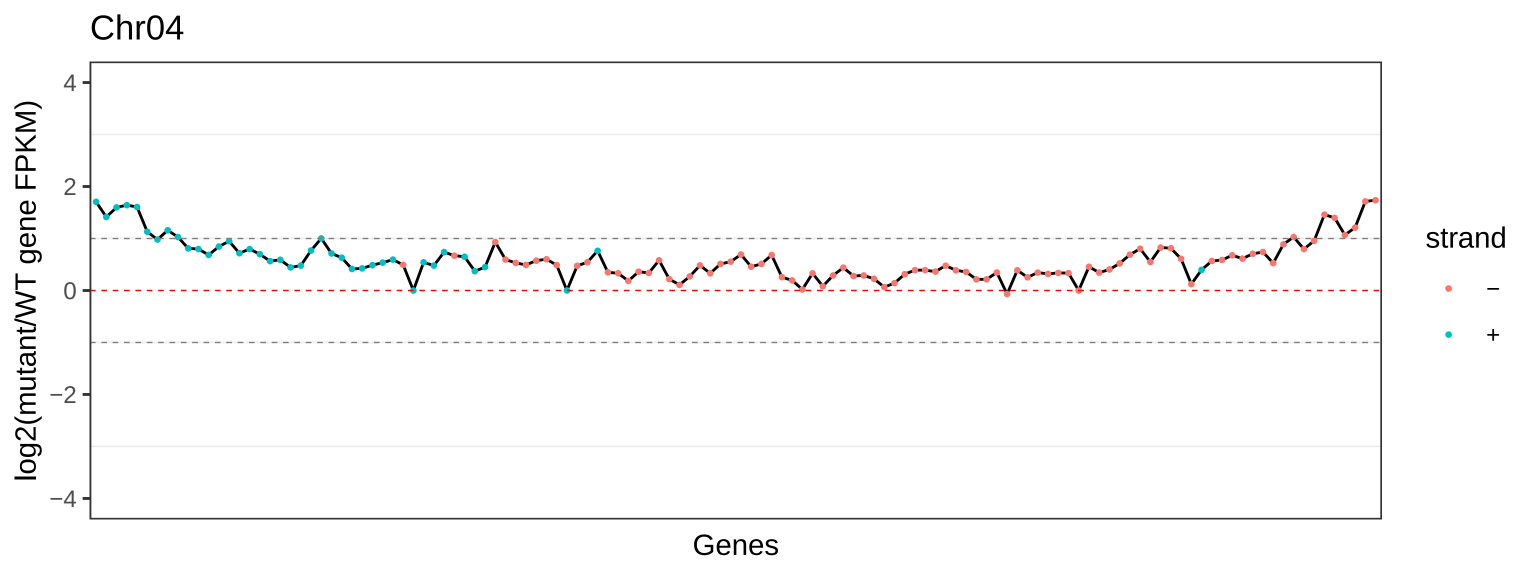
Chr04
log2(mutant/WT gene FPKM)
4
2
0
−2
−4
Genes
strand
−
+

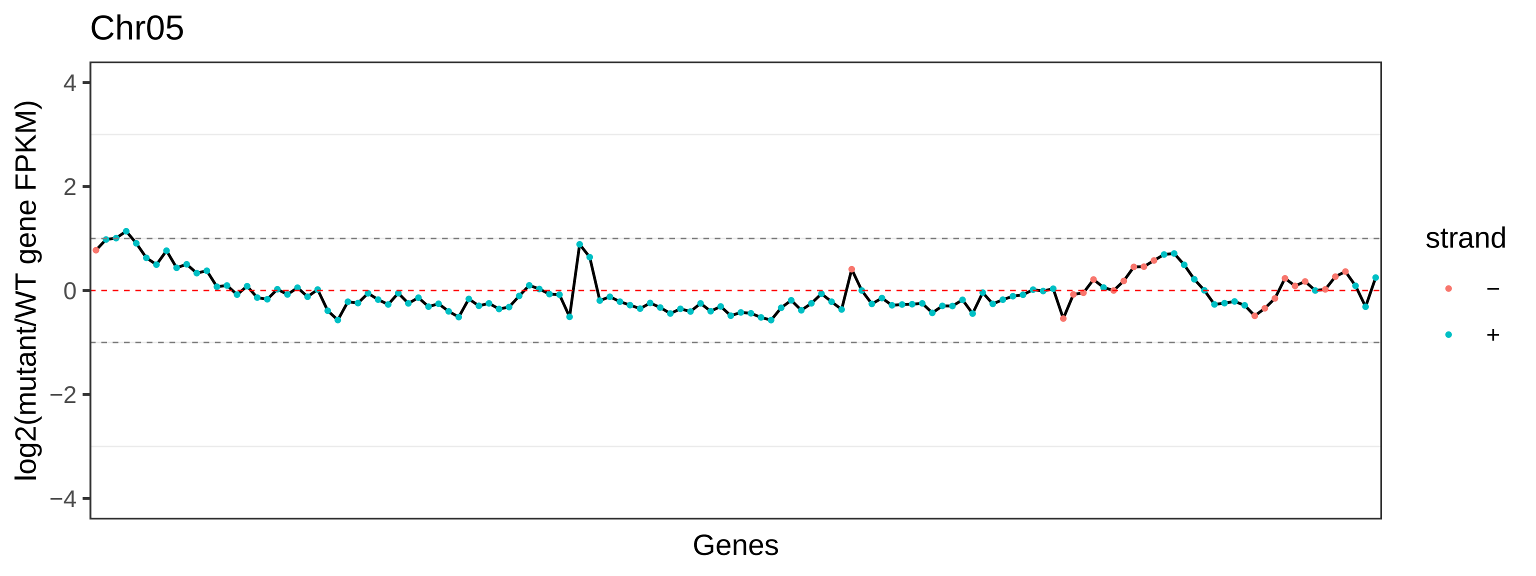

Chr05
4
2
0
−2
−4
log2(mutant/WT gene FPKM)
Genes
strand
−
+

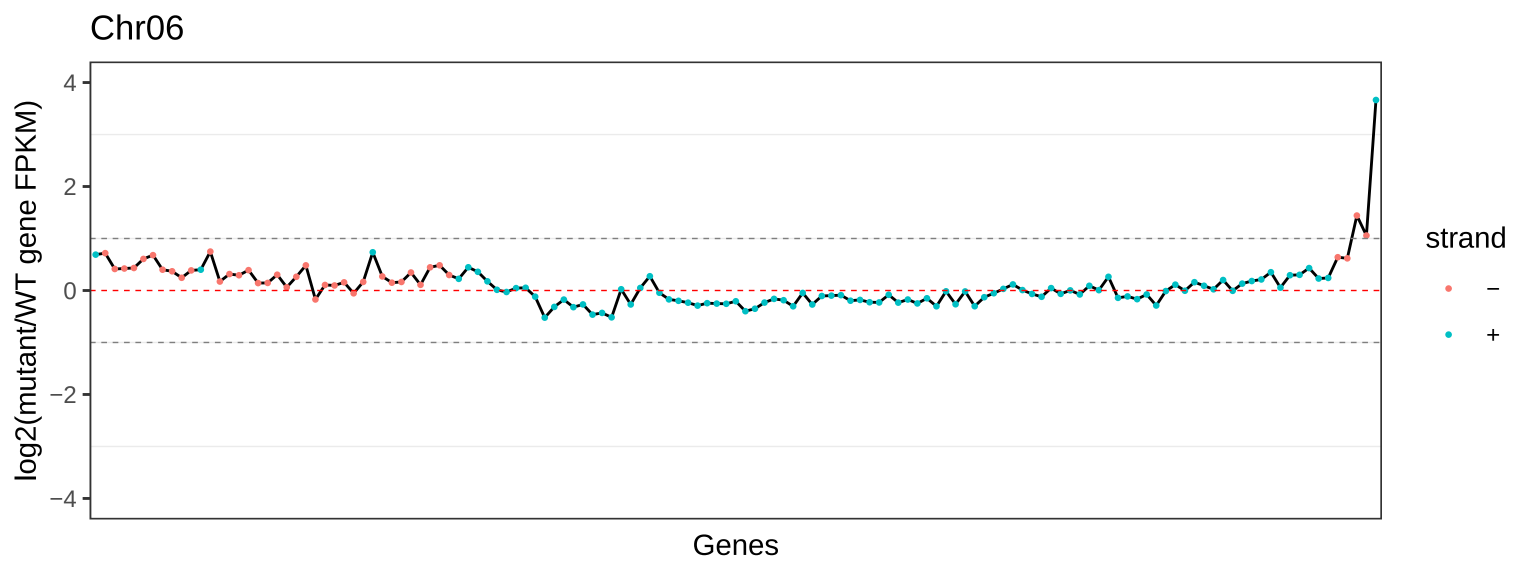

Chr06
log2(mutant/WT gene FPKM)
4
2
0
−2
−4
Genes
strand
−
+

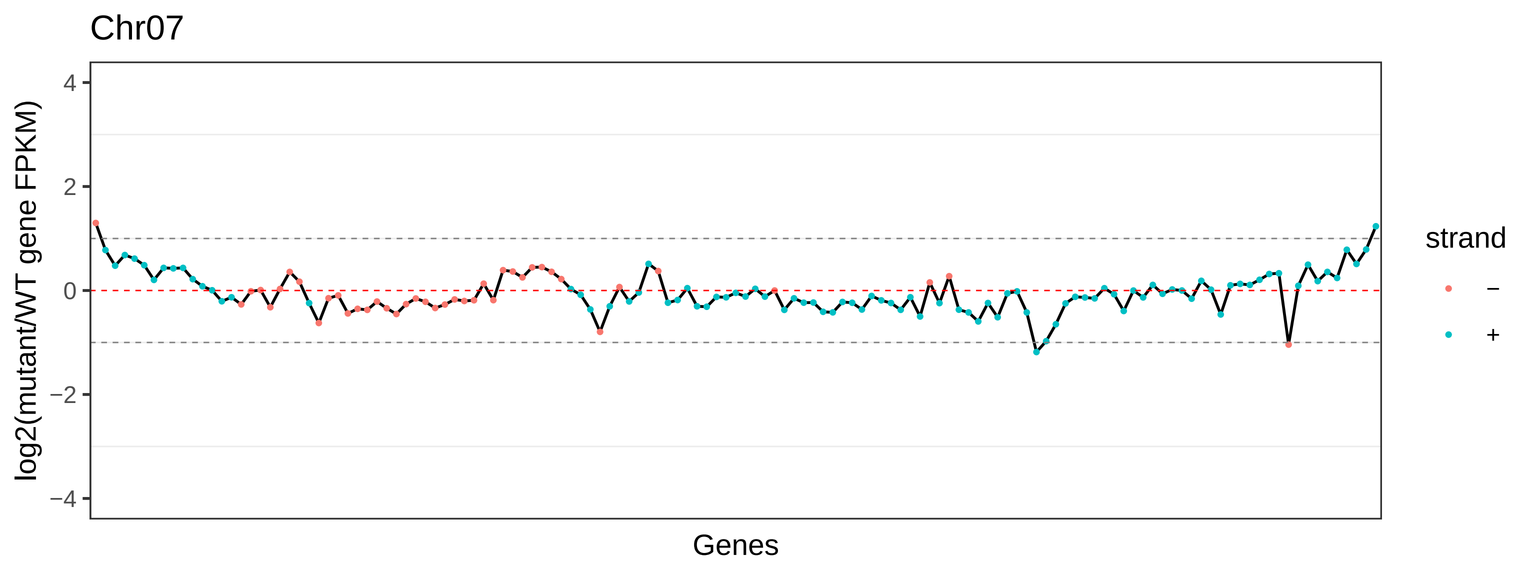

Chr07
4
2
0
−2
−4
log2(mutant/WT gene FPKM)
Genes
strand
−
+

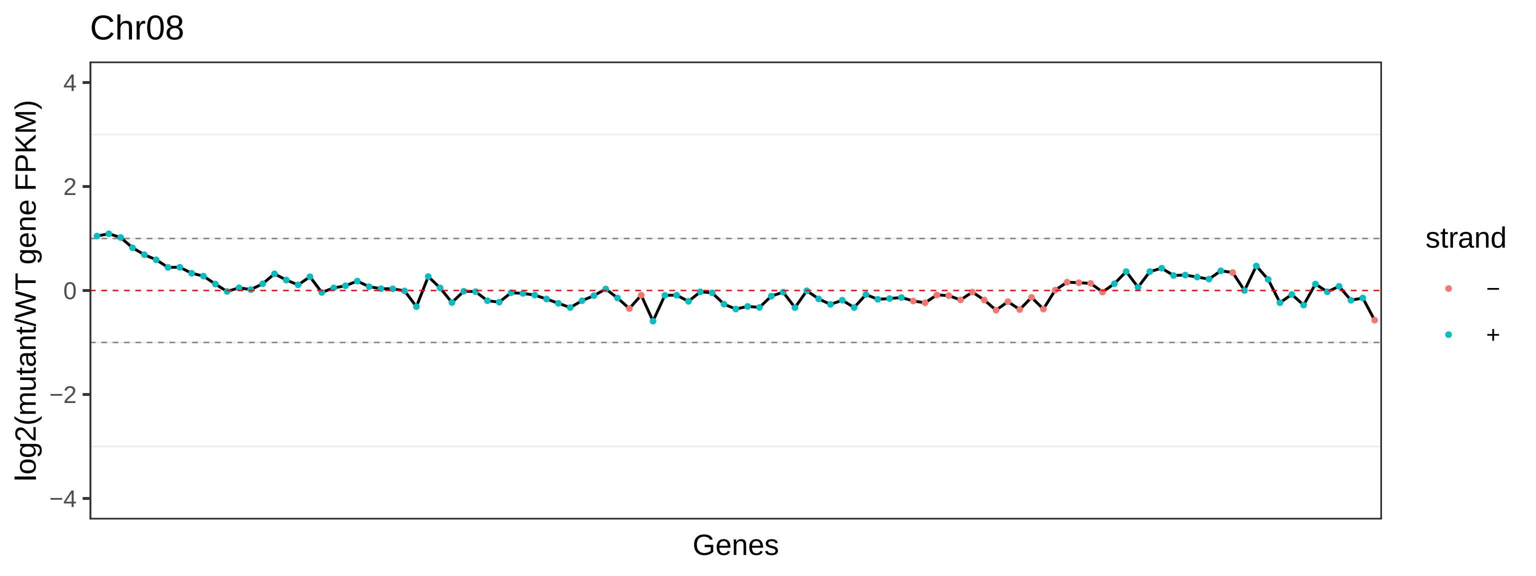

Chr08
log2(mutant/WT gene FPKM)
4
2
0
−2
−4
Genes
strand
−
+

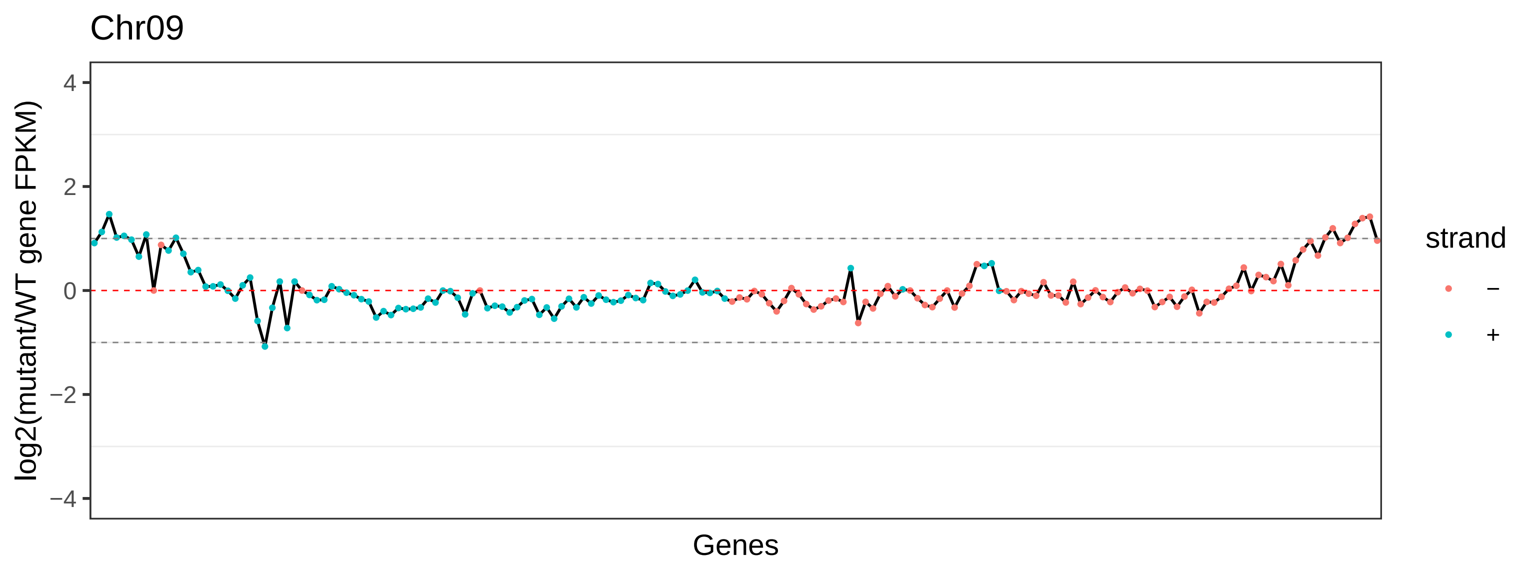

Chr09
log2(mutant/WT gene FPKM)
4
2
0
−2
−4
Genes
strand
−
+

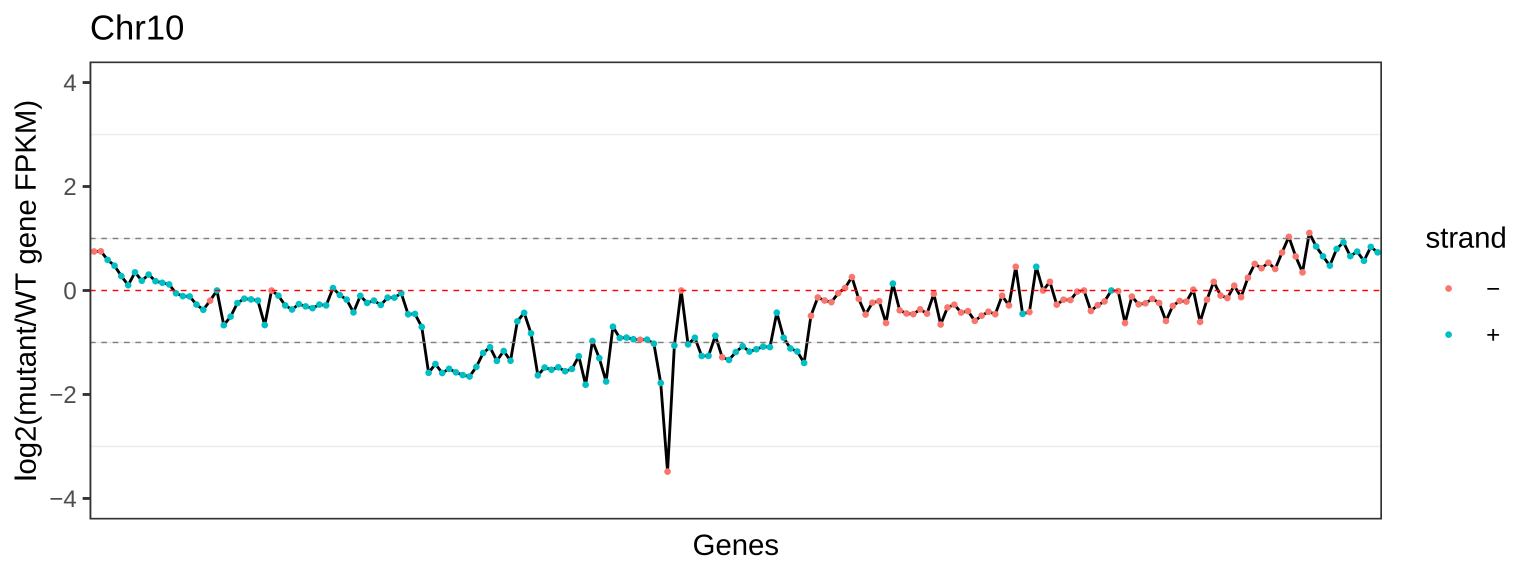
Chr10
log2(mutant/WT gene FPKM)
4
2
0
−2
−4
Genes
strand
−
+

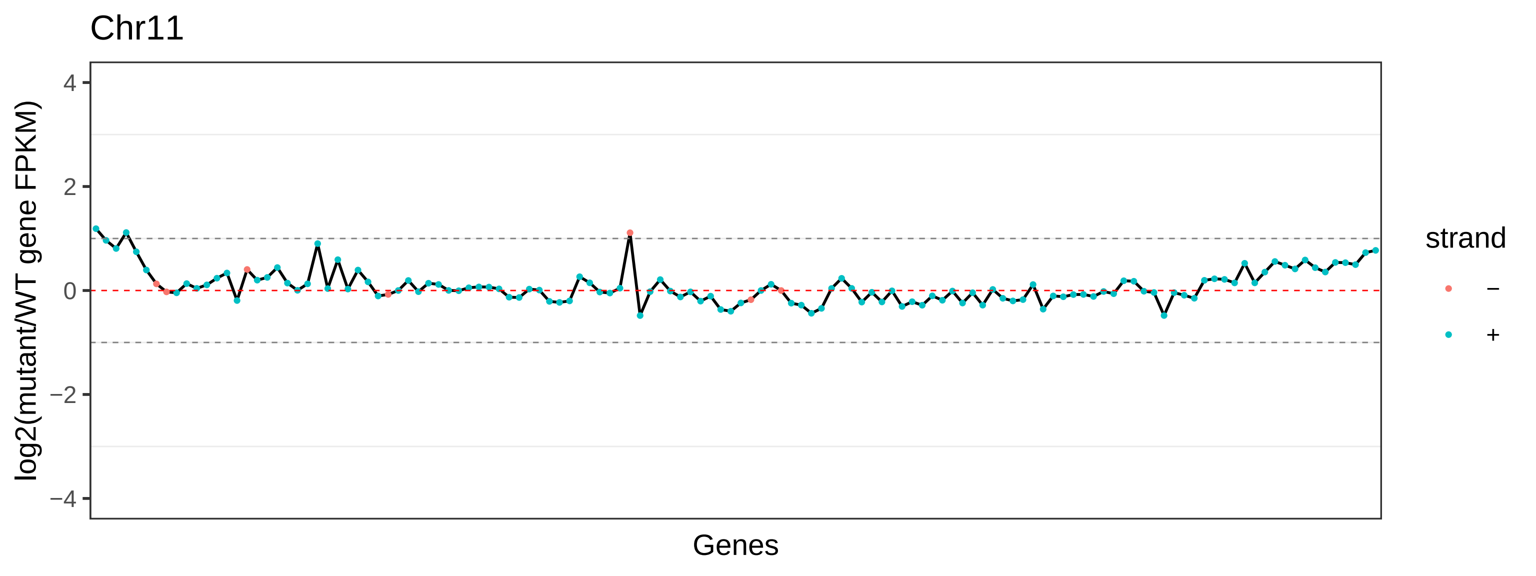

Chr11
log2(mutant/WT gene FPKM)
4
2
0
−2
−4
Genes
strand
−
+

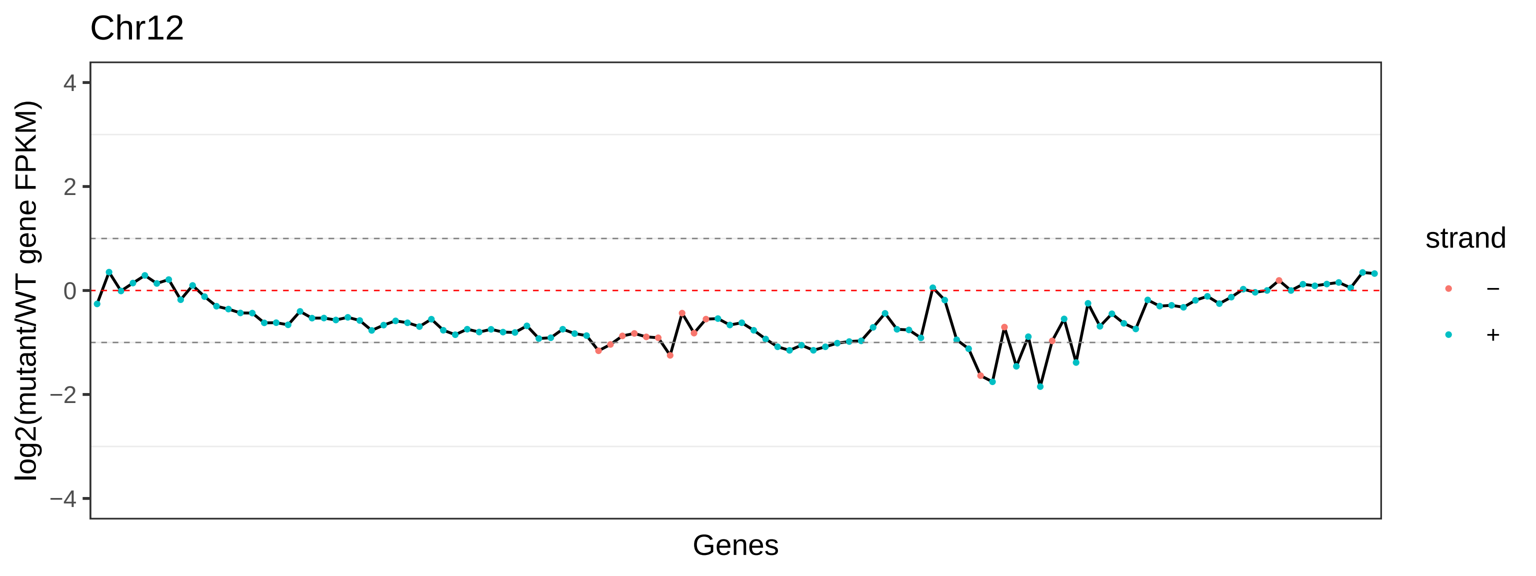

Chr12
log2(mutant/WT gene FPKM)
4
2
0
−2
−4
Genes
strand
−
+

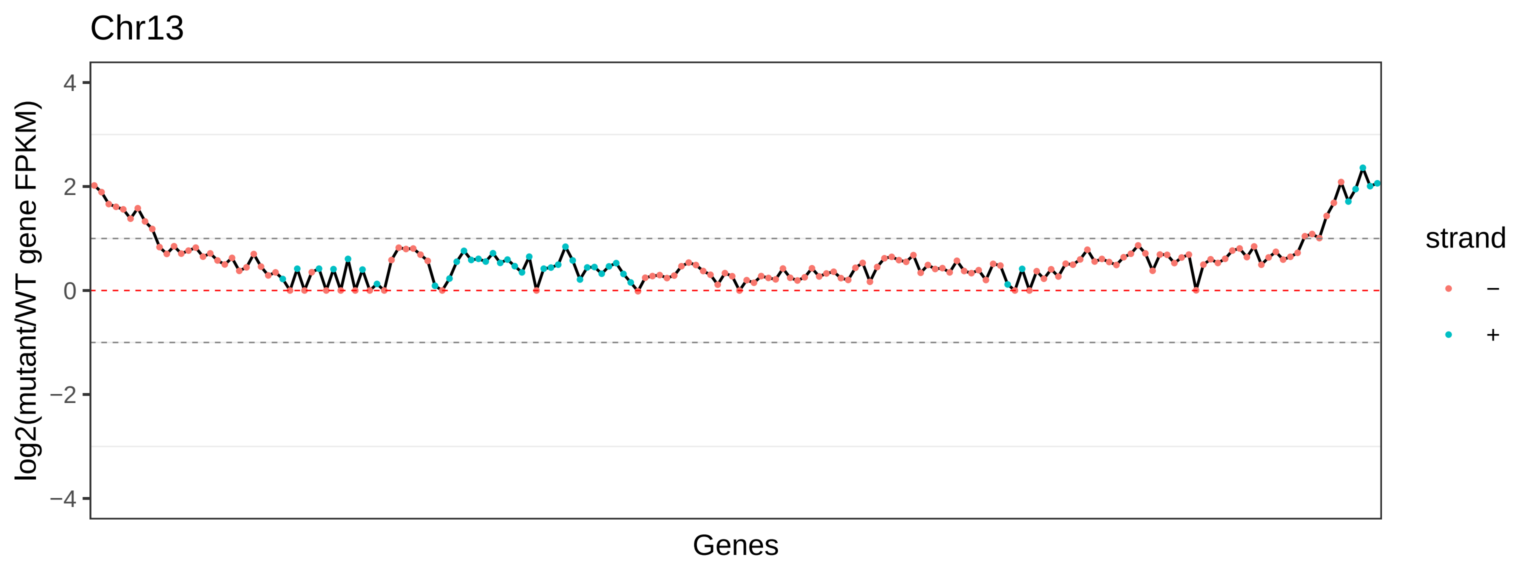
Chr13
log2(mutant/WT gene FPKM)
4
2
0
−2
−4
Genes
strand
−
+

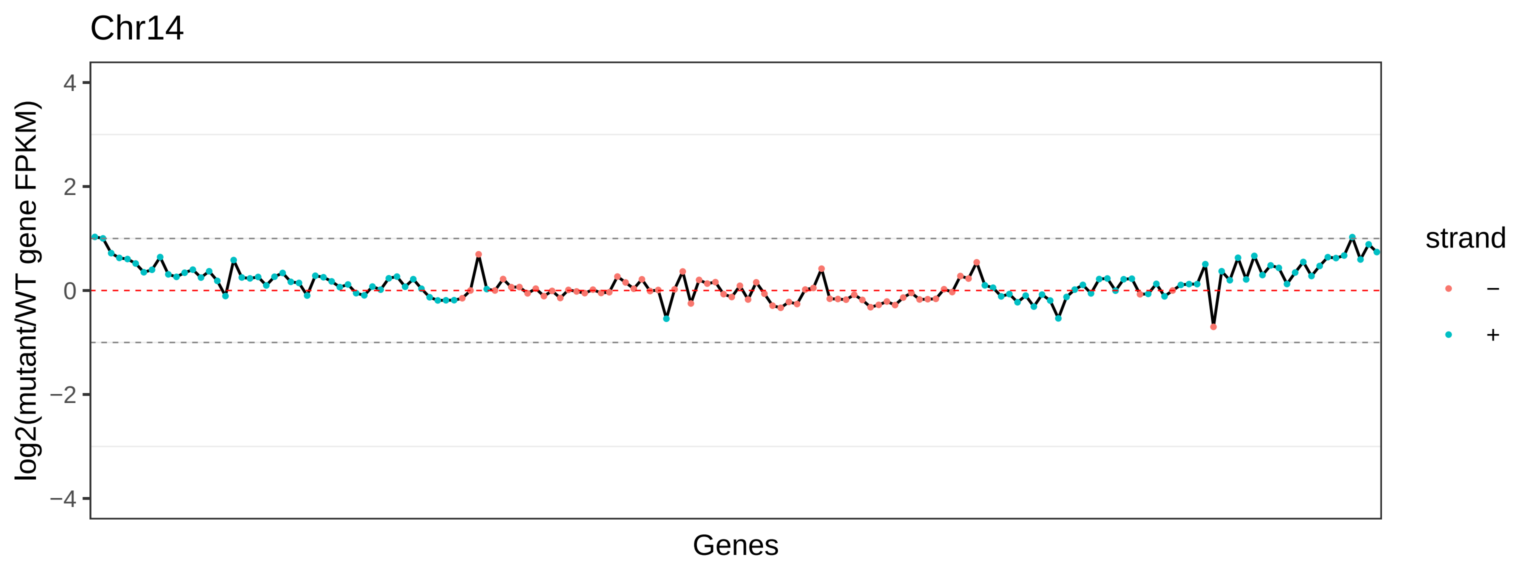

Chr14
log2(mutant/WT gene FPKM)
4
2
0
−2
−4
Genes
strand
−
+

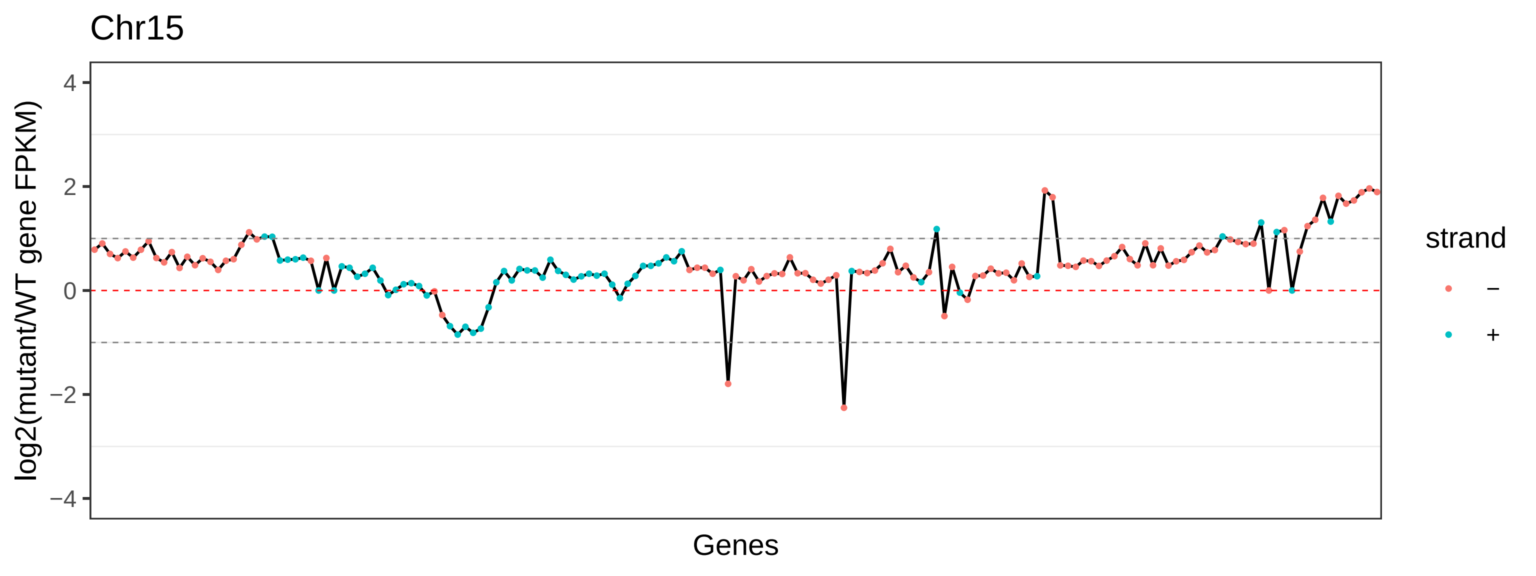
Chr15
log2(mutant/WT gene FPKM)
4
2
0
−2
−4
Genes
strand
−
+

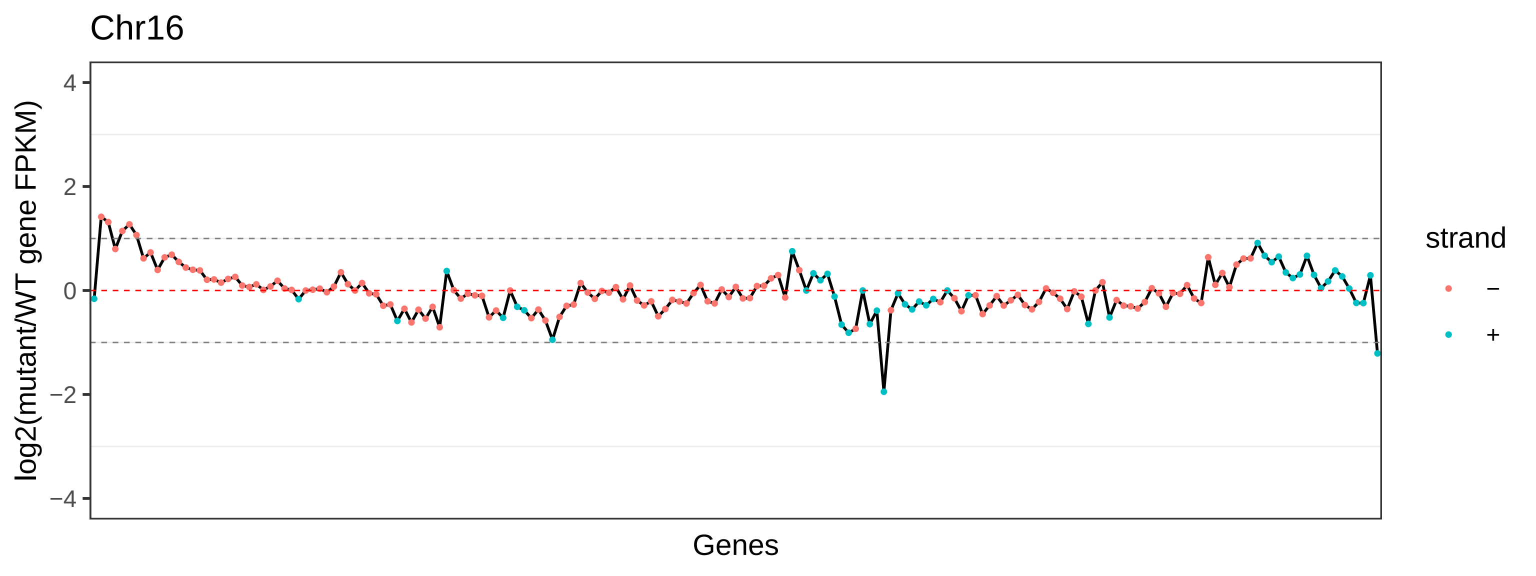

Chr16
log2(mutant/WT gene FPKM)
4
2
0
−2
−4
Genes
strand
−
+

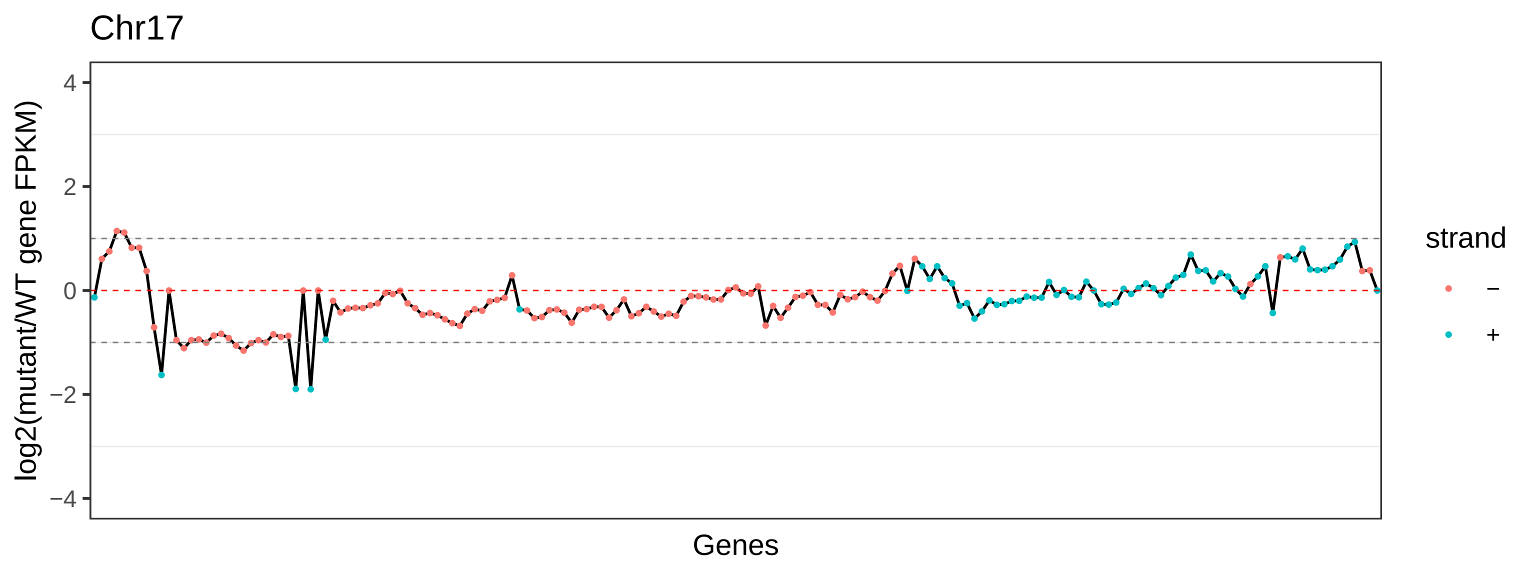
Chr17
4
2
0
−2
−4
log2(mutant/WT gene FPKM)
Genes
strand
−
+

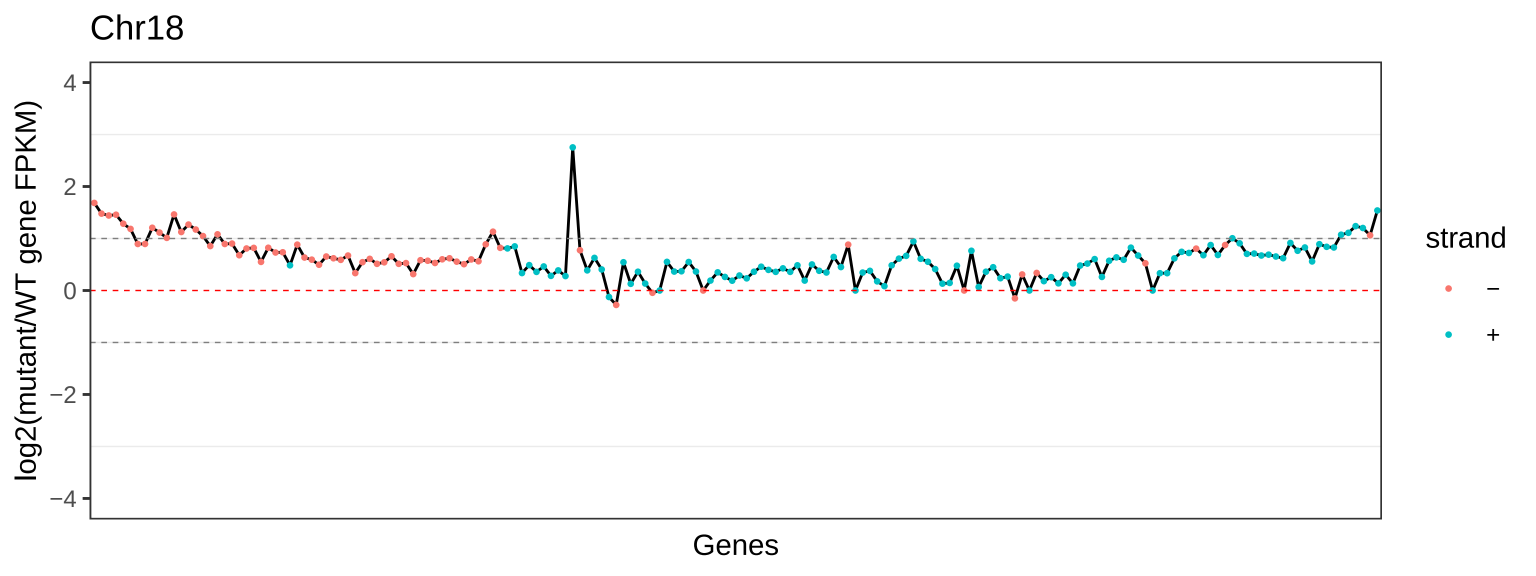

Chr18
log2(mutant/WT gene FPKM)
4
2
0
−2
−4
Genes
strand
−
+

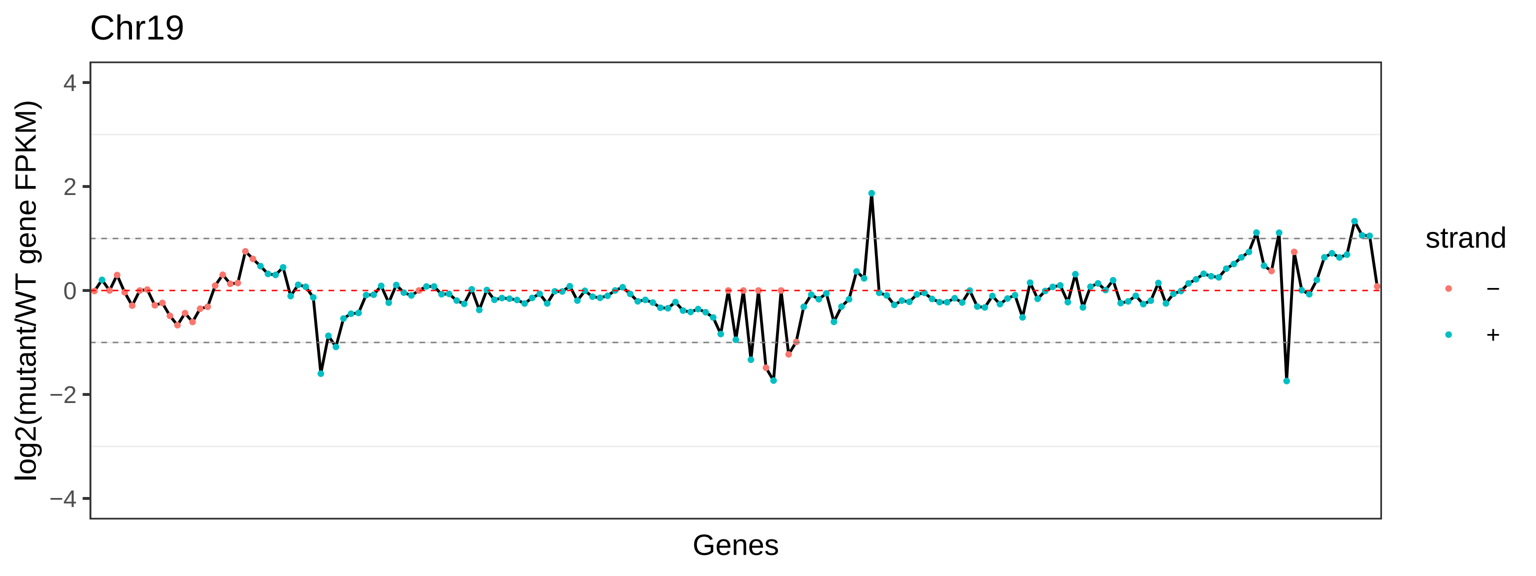
Chr19
log2(mutant/WT gene FPKM)
4
2
0
−2
−4
Genes
strand
−
+

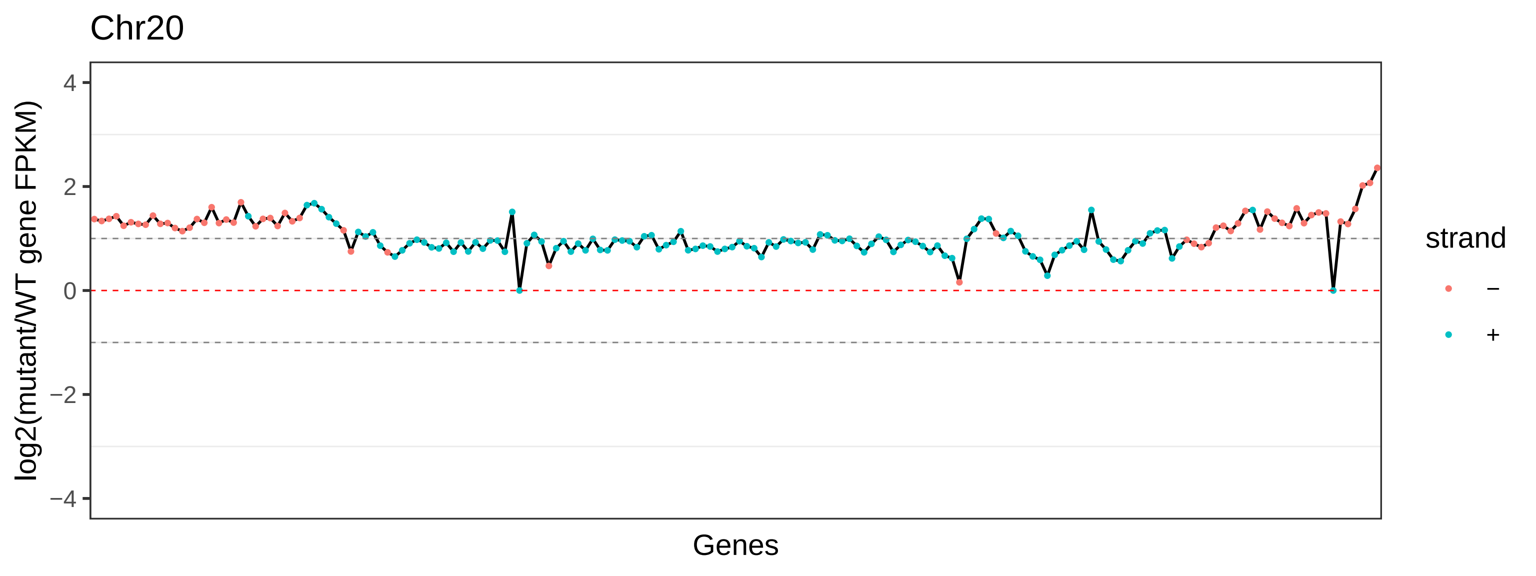

Chr20
log2(mutant/WT gene FPKM)
4
2
0
−2
−4
Genes
strand
−
+

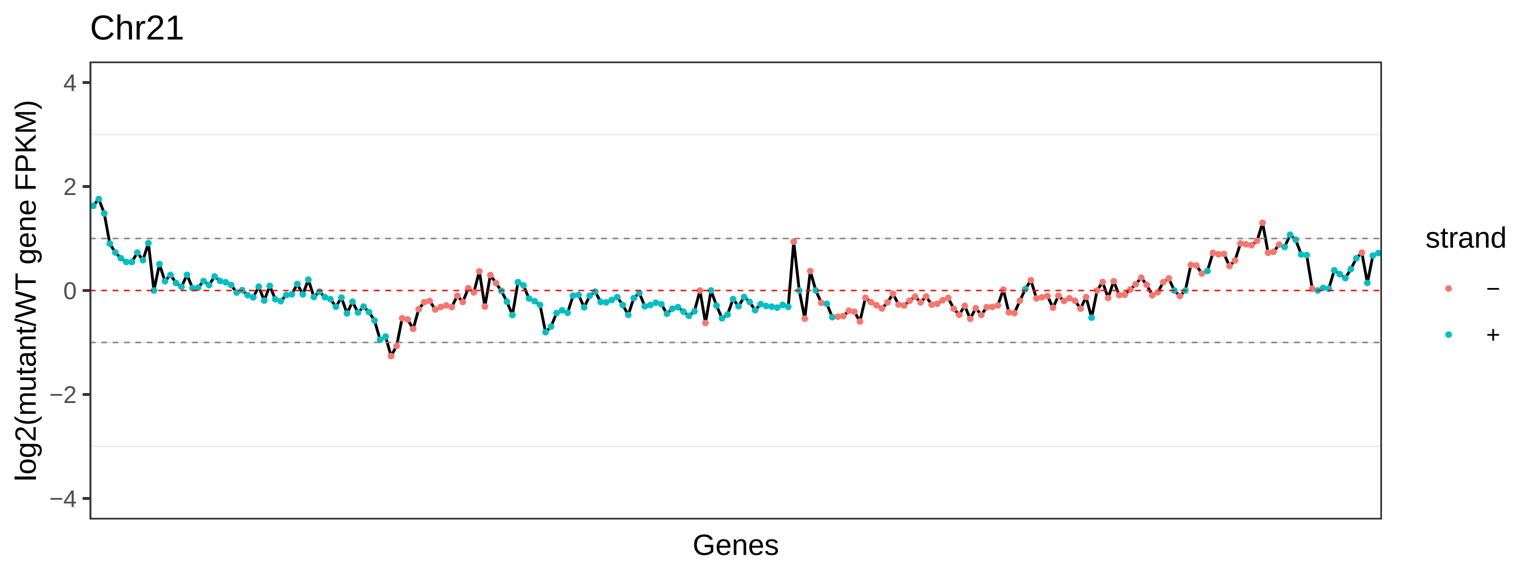
Chr21
log2(mutant/WT gene FPKM)
4
2
0
−2
−4
Genes
strand
−
+

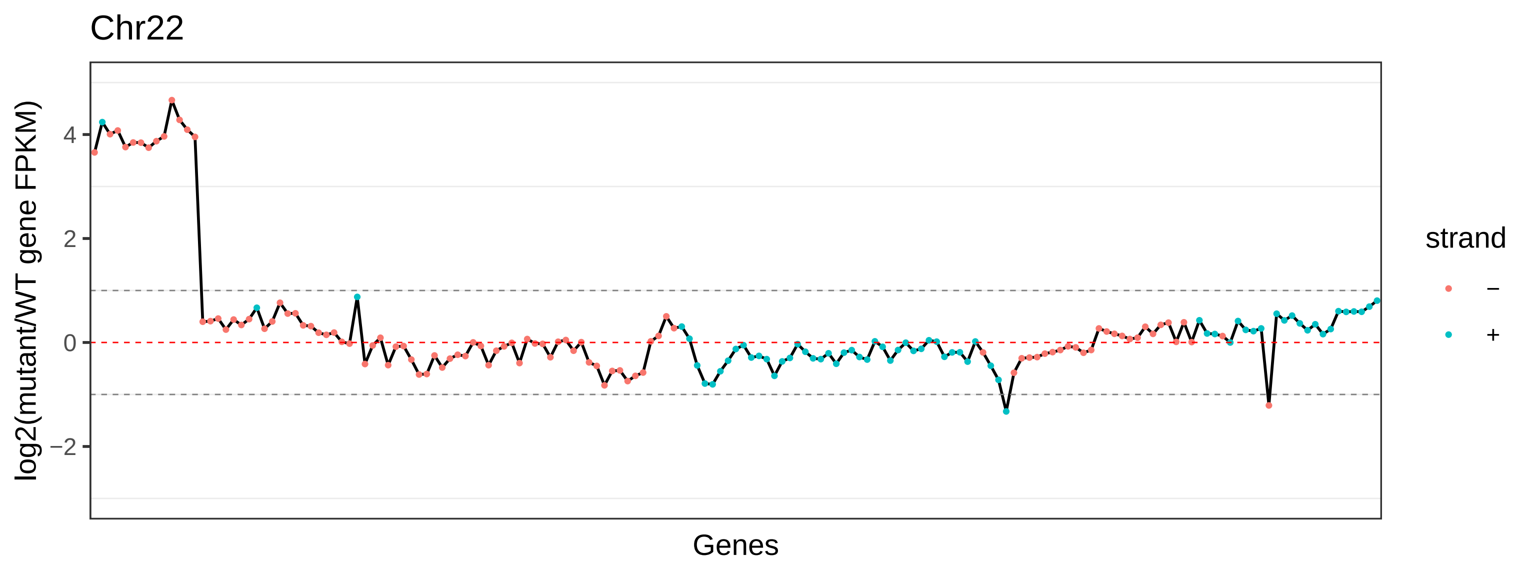
Chr22
log2(mutant/WT gene FPKM)
4
2
0
−2
Genes
strand
−
+

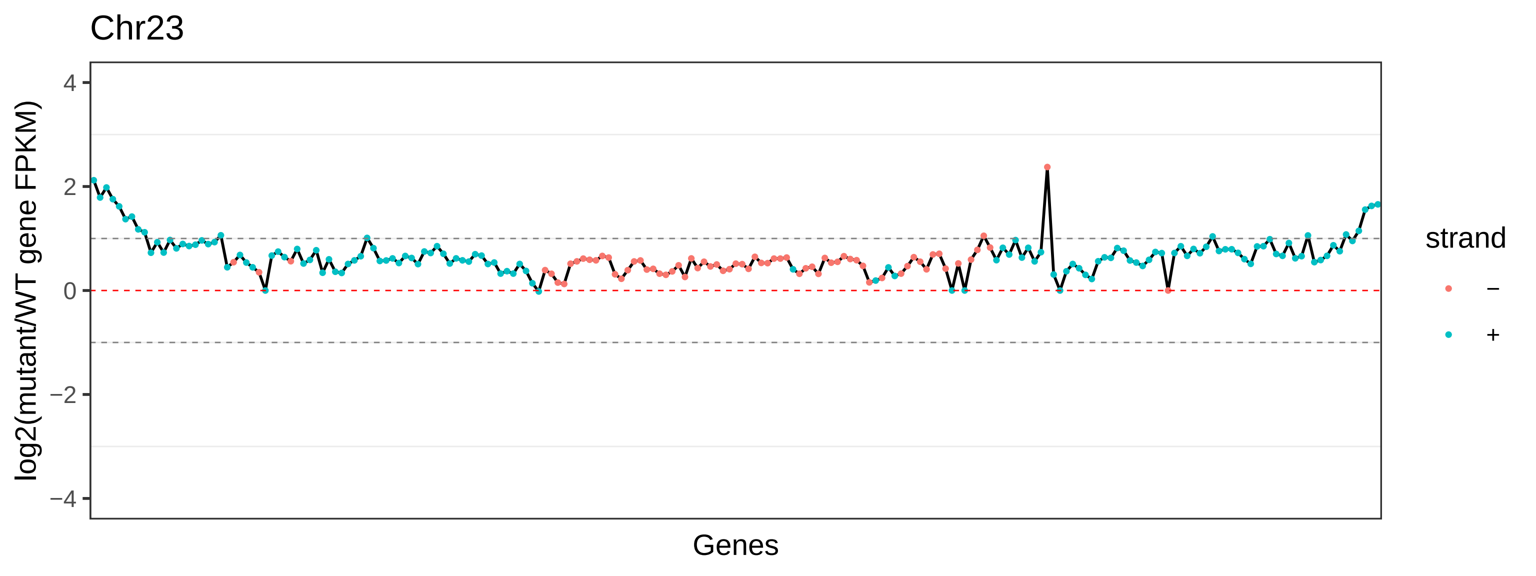

Chr23
log2(mutant/WT gene FPKM)
4
2
0
−2
−4
Genes
strand
−
+

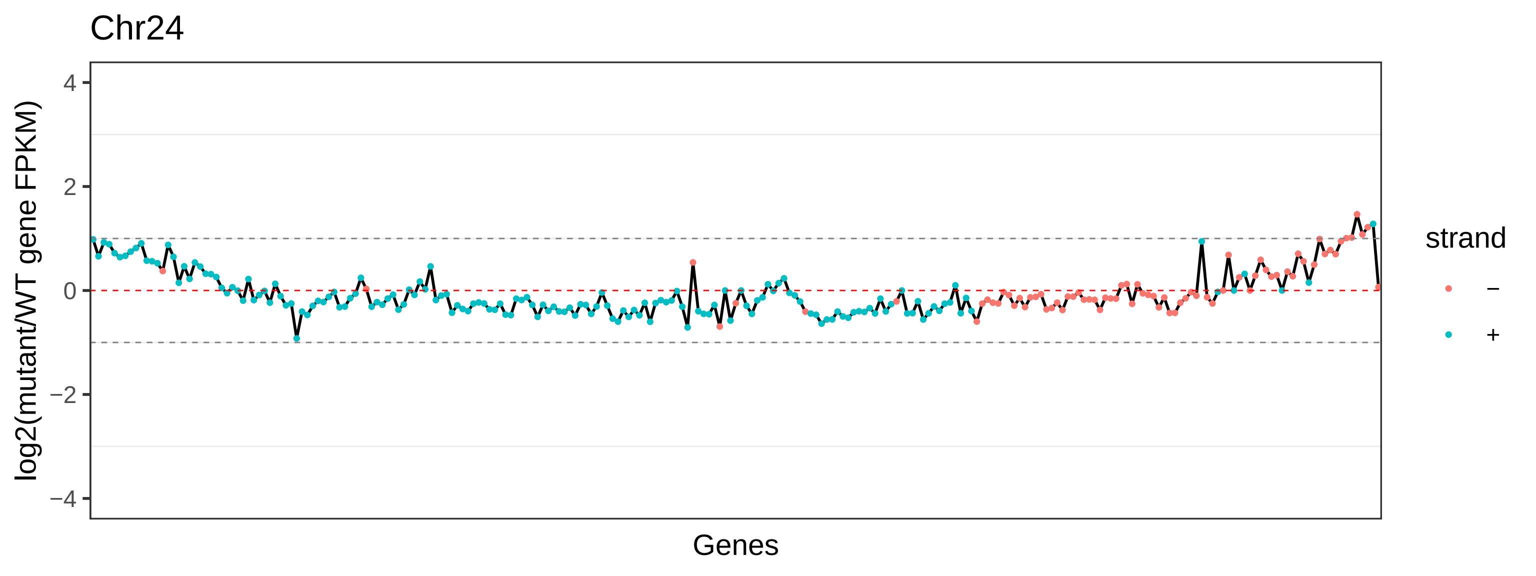
Chr24
4
2
0
−2
−4
log2(mutant/WT gene FPKM)
Genes
strand
−
+

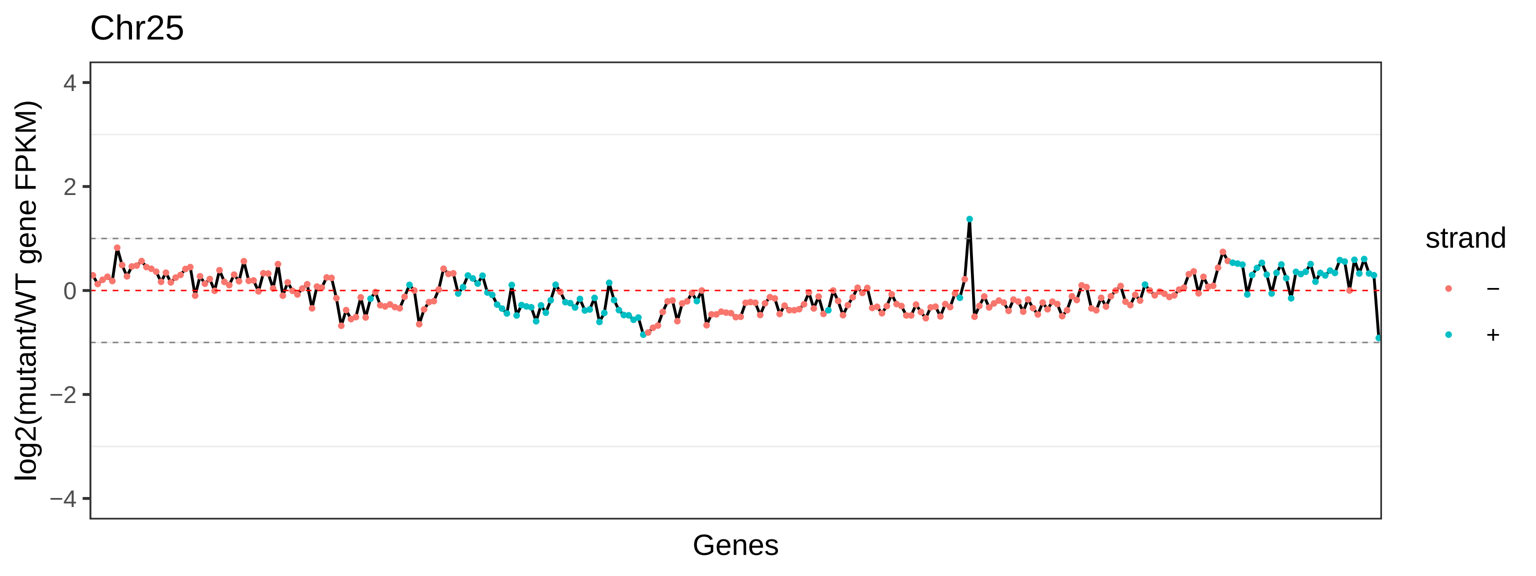

Chr25
log2(mutant/WT gene FPKM)
4
2
0
−2
−4
Genes
strand
−
+

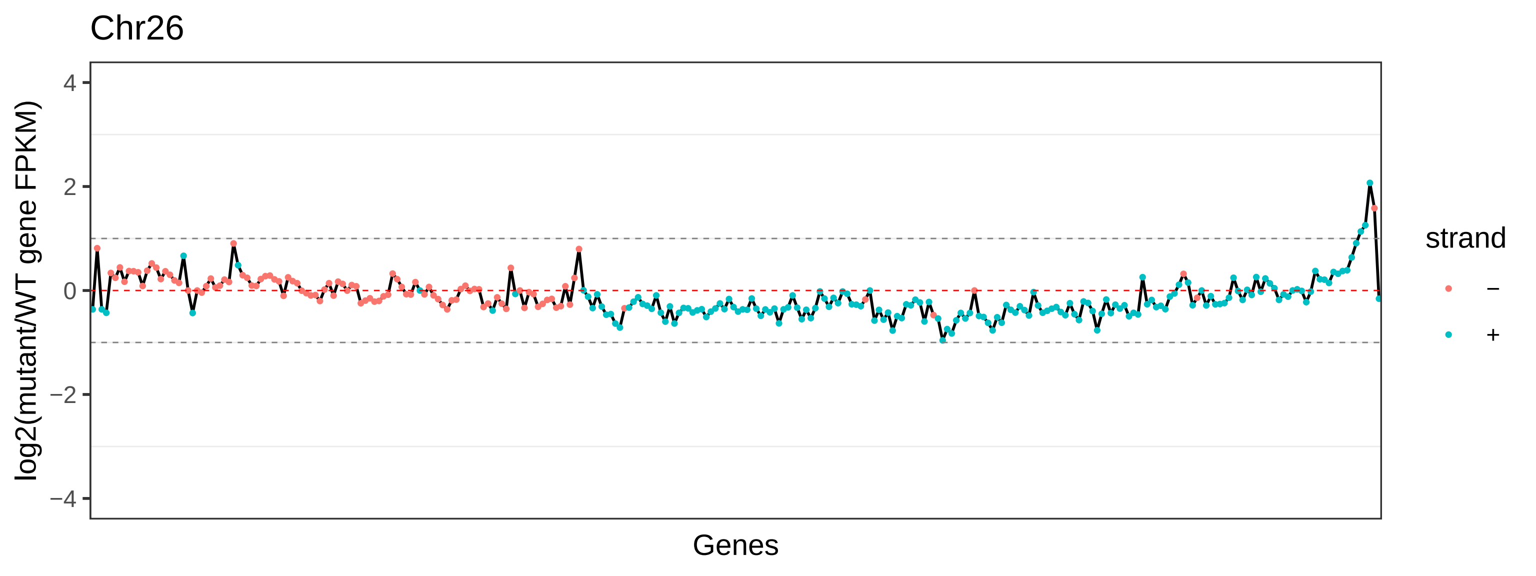

Chr26
log2(mutant/WT gene FPKM)
4
2
0
−2
−4
Genes
strand
−
+

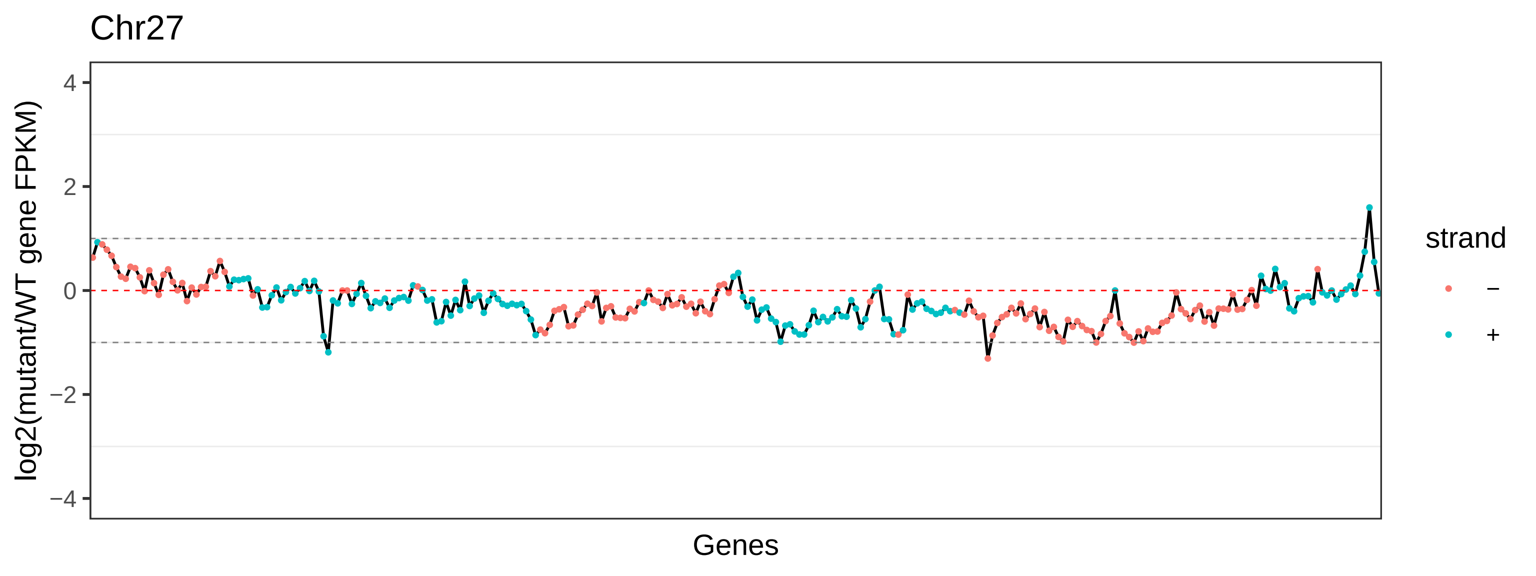
Chr27
log2(mutant/WT gene FPKM)
4
2
0
−2
−4
Genes
strand
−
+

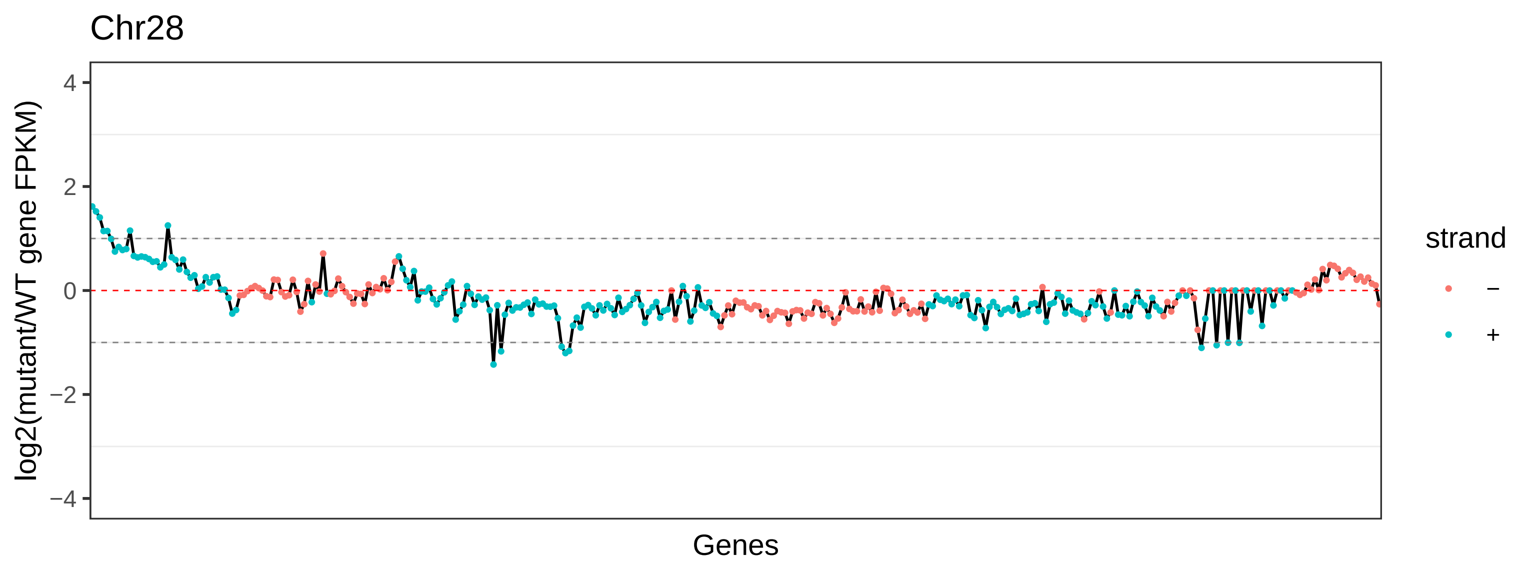

Chr28
log2(mutant/WT gene FPKM)
4
2
0
−2
−4
Genes
strand
−
+

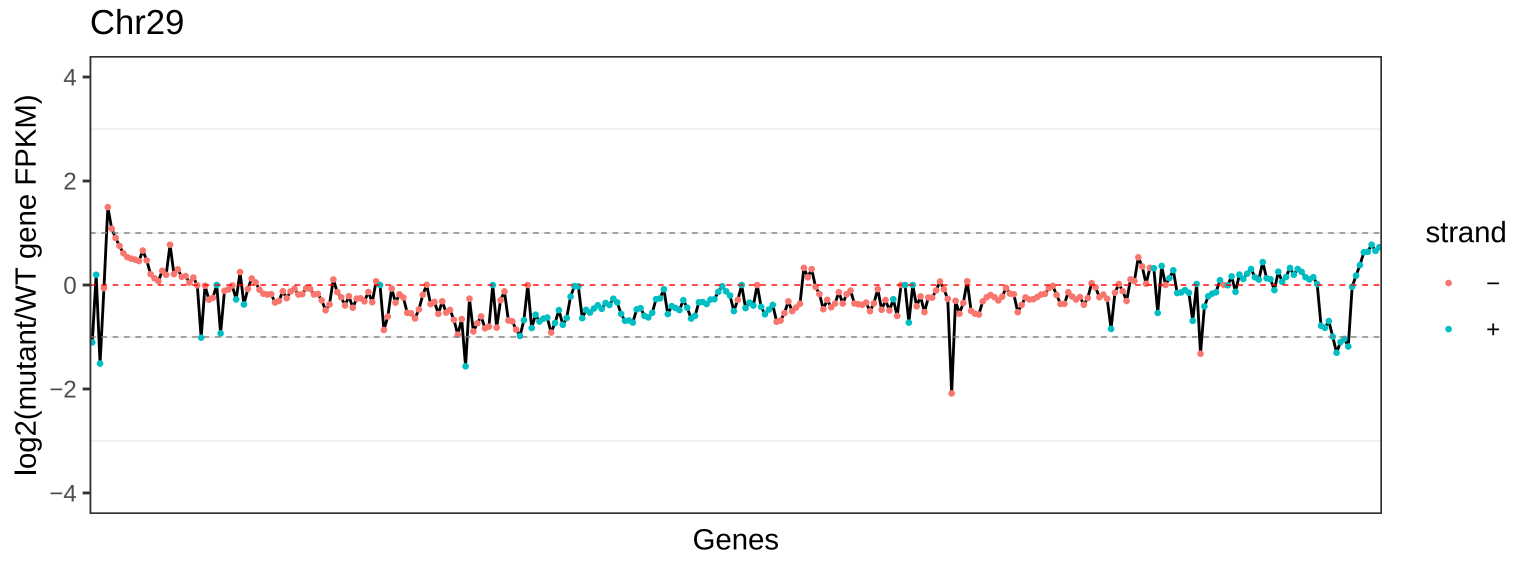
Chr29
4
2
0
−2
−4
log2(mutant/WT gene FPKM)
Genes
strand
−
+

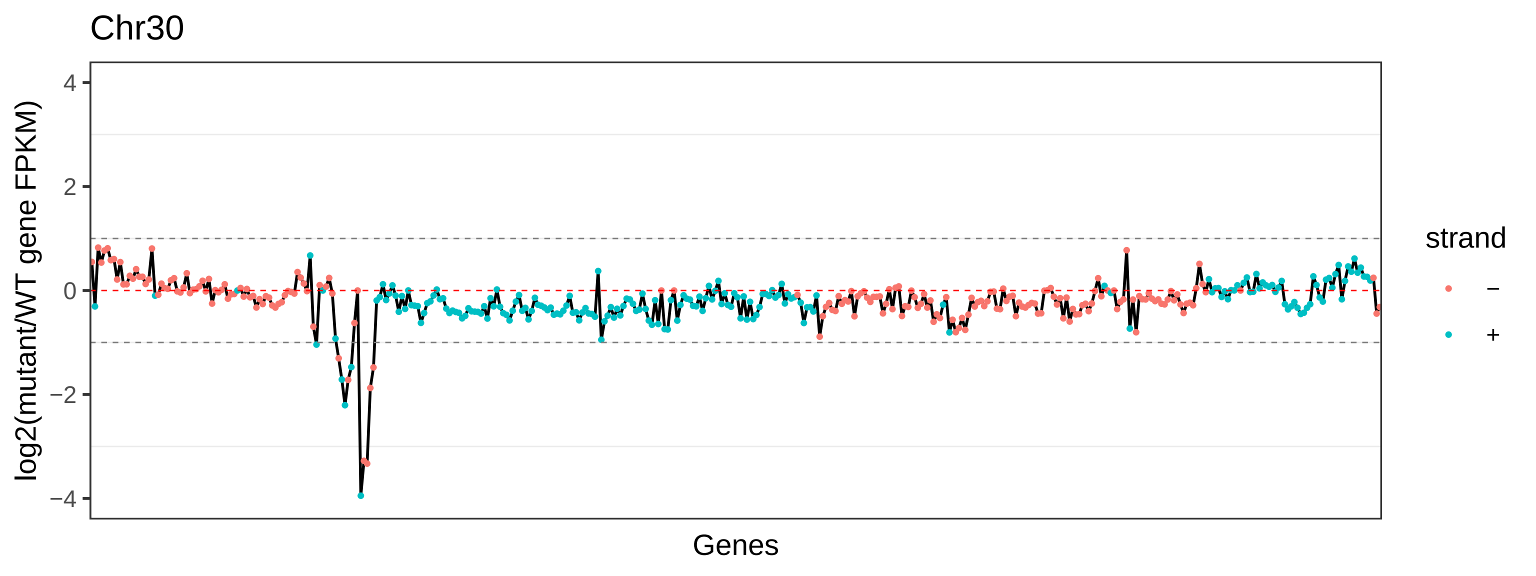
Chr30
log2(mutant/WT gene FPKM)
4
2
0
−2
−4
Genes
strand
−
+

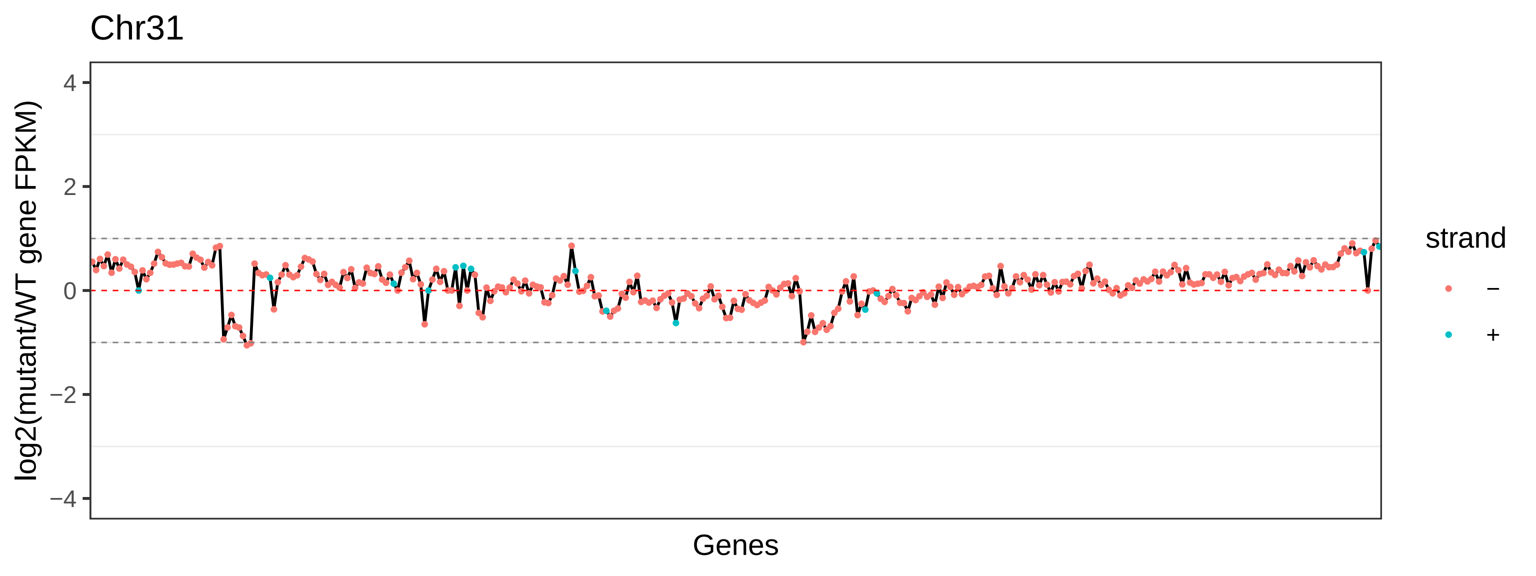

Chr31
log2(mutant/WT gene FPKM)
4
2
0
−2
−4
Genes
strand
−
+

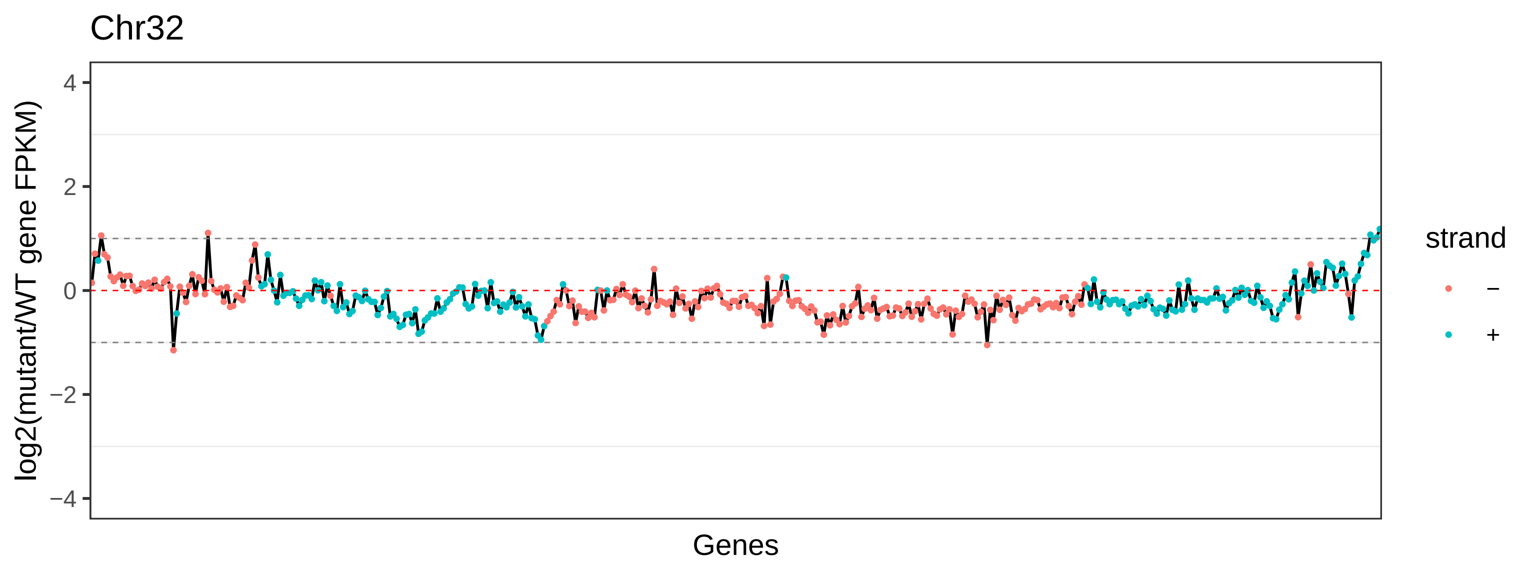

Chr32
log2(mutant/WT gene FPKM)
4
2
0
−2
−4
Genes
strand
−
+

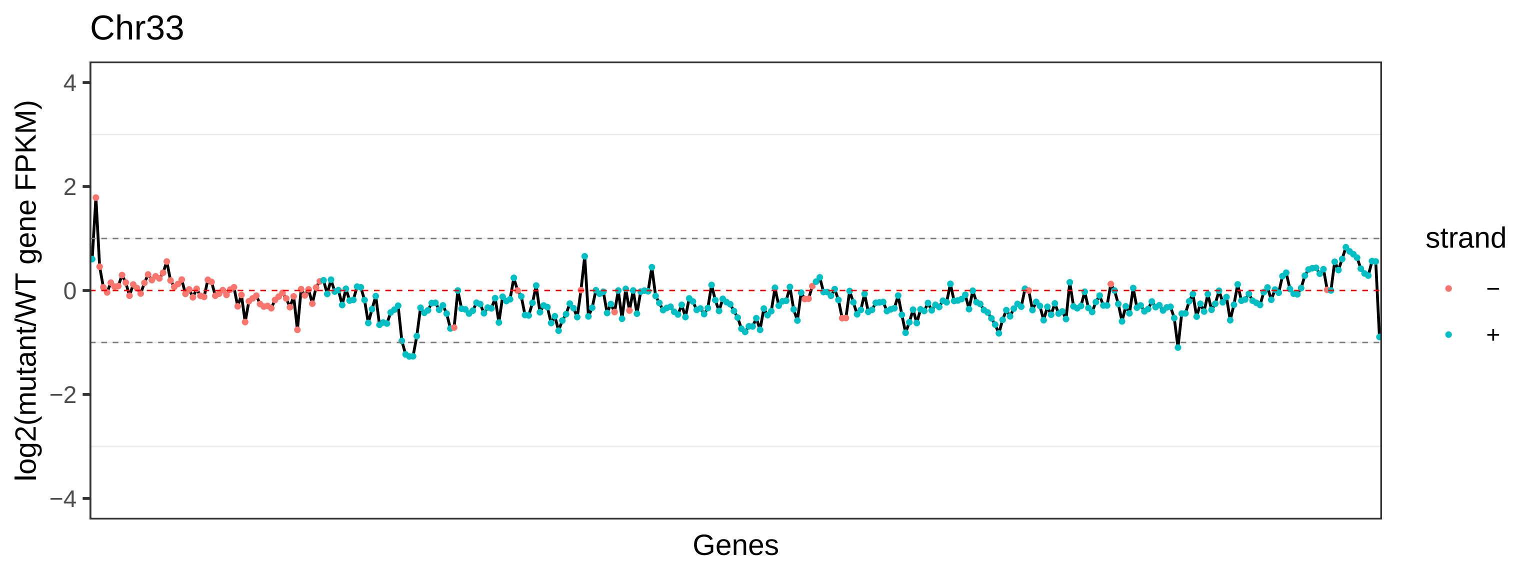
Chr33
log2(mutant/WT gene FPKM)
4
2
0
−2
−4
Genes
strand
−
+

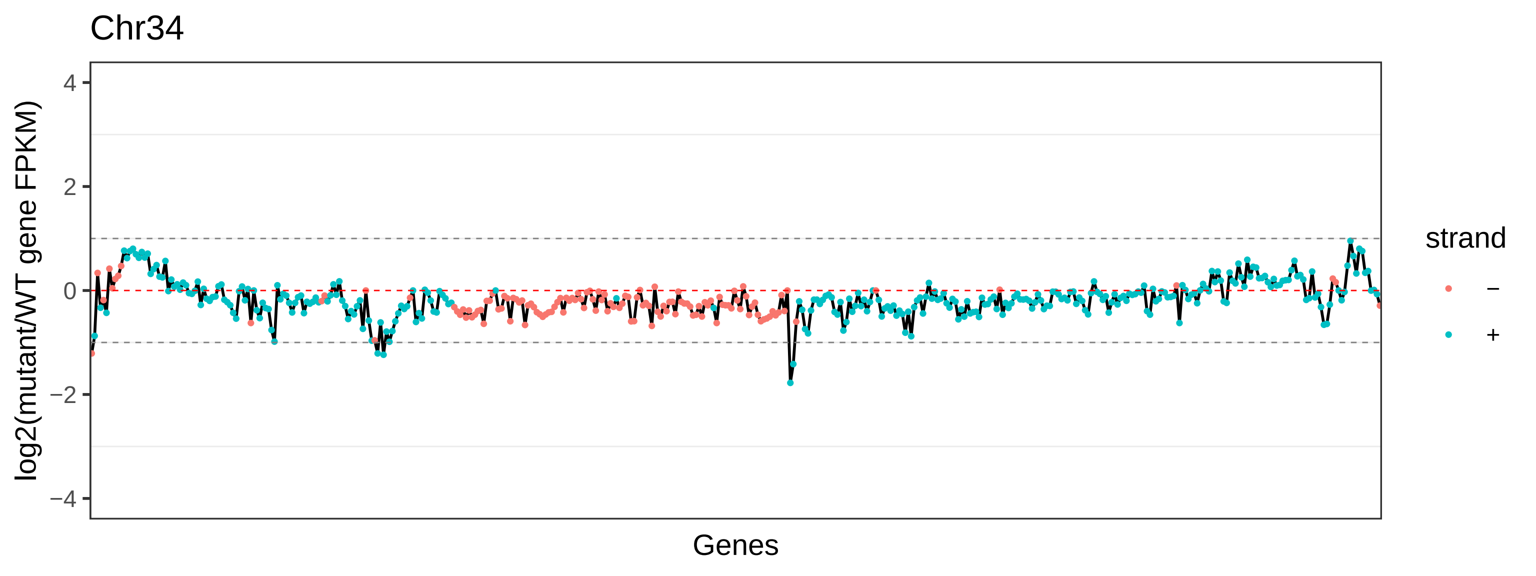
Chr34
log2(mutant/WT gene FPKM)
4
2
0
−2
−4
Genes
strand
−
+

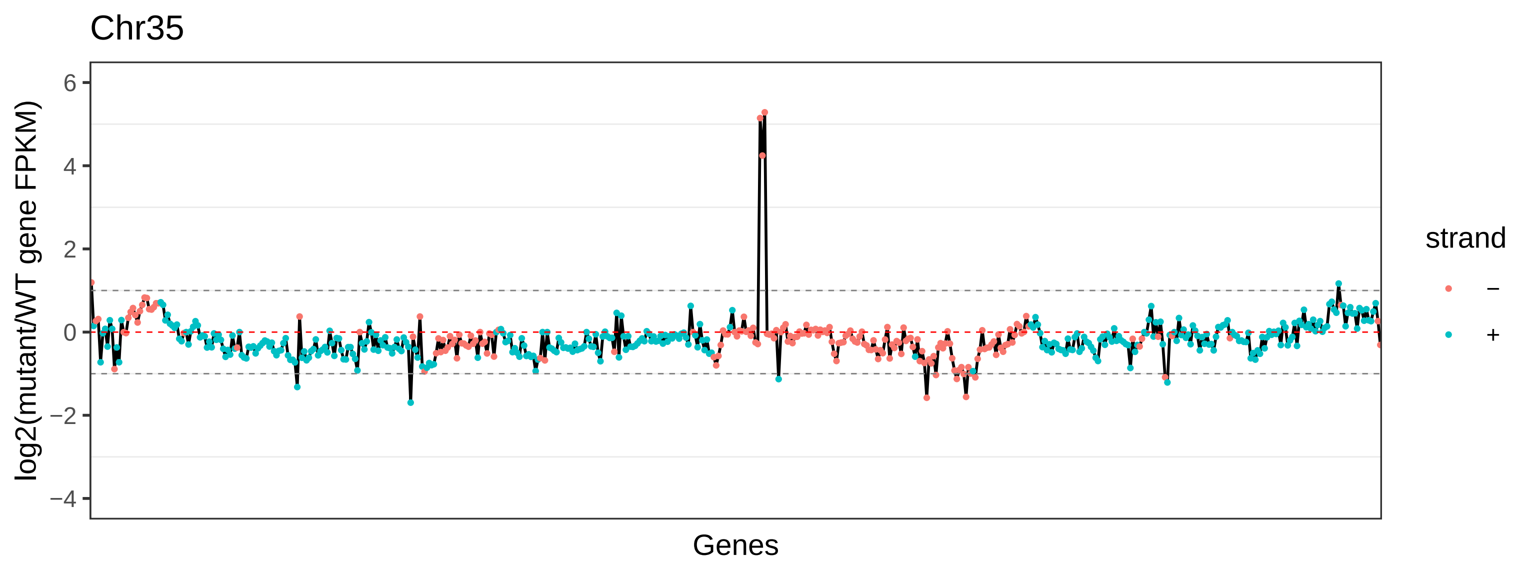
Chr35
6
4
2
0
−2
−4
log2(mutant/WT gene FPKM)
Genes
strand
−
+

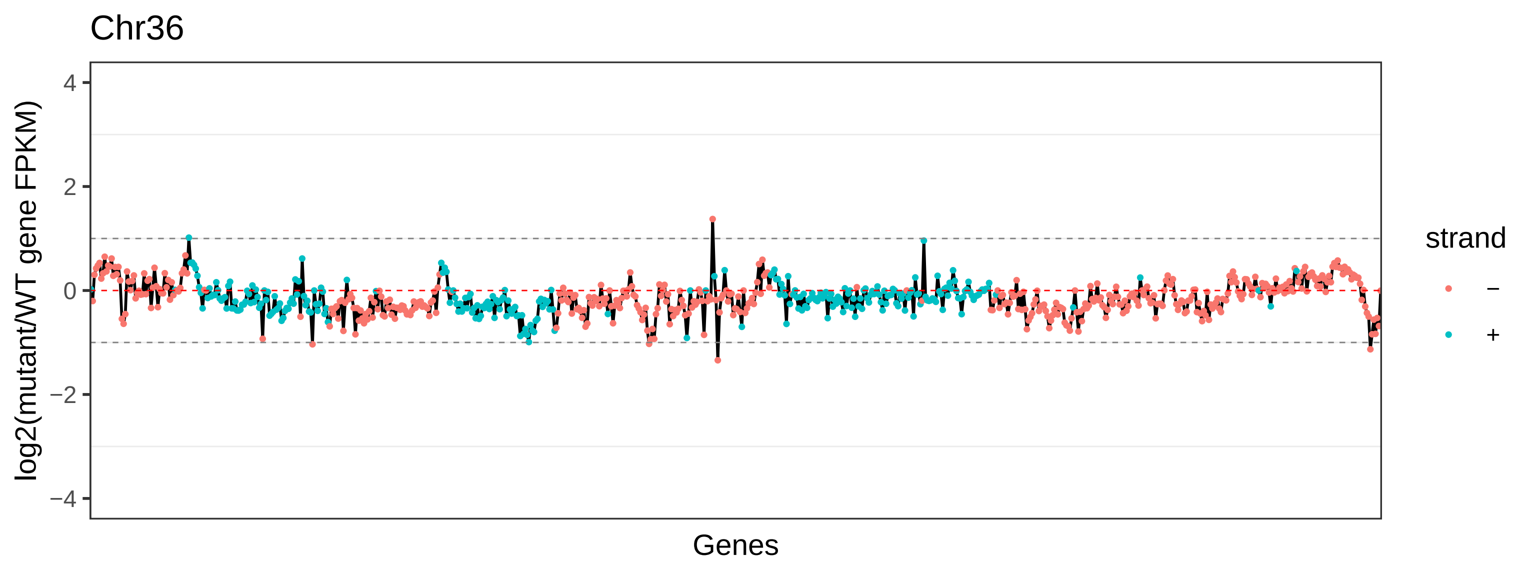
Chr36
log2(mutant/WT gene FPKM)
4
2
0
−2
−4
Genes
strand
−
+
