# Supplementary figures and images for: Decreased glutamate transport in acivicin resistant Leishmania tarentolae
Source: PLoS Negl Trop Dis. 2021 Dec 16;15(12):e0010046. doi: 10.1371/journal.pntd.0010046 (PMC8718007; doi:10.1371/journal.pntd.0010046)

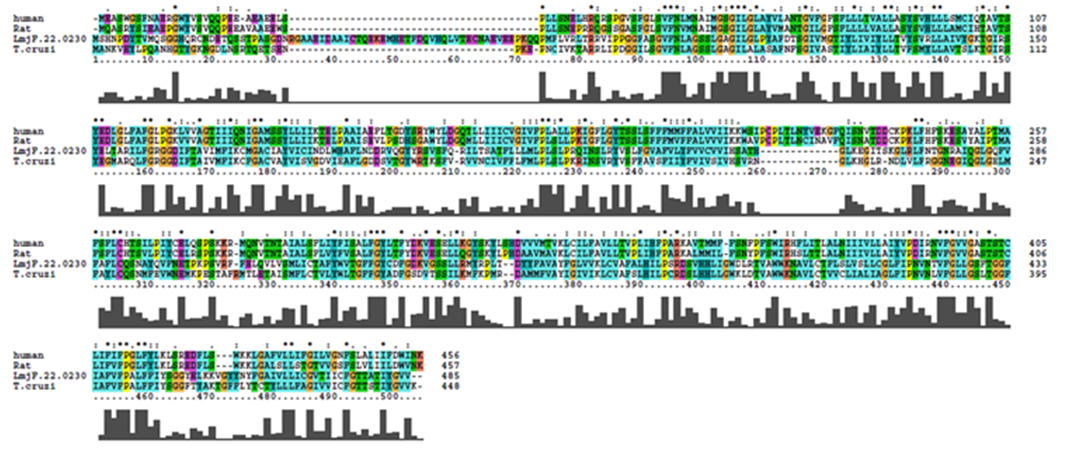

Supplement: S2 Fig — Alignment of the Homo sapiens solute carrier family 38 member 6 (SLC38A6), Trypanosoma cruzi strain CL Brener gene TcCLB.507811.100, Rattus norvegicus solute carrier family 38 member 6 (Slc38a6) and L. major strain Friedlin gene LmjF22.0230. Blue represents residues A, C, F, I, M, V and W; Dark Blue represents residues H and Y; Pink represents residues E and D; Green represents residues N, Q, S and T; Yellow represents residue P; Orange represents residue G; Coral represents residues K and R; ‘*’ indicates position which have a single fully conserved residue; ‘:’indicates a strong group of conserved amino acids; ‘.’ indicates a weaker group of conserved amino acids. (TIF) [file pntd.0010046.s002.tif]

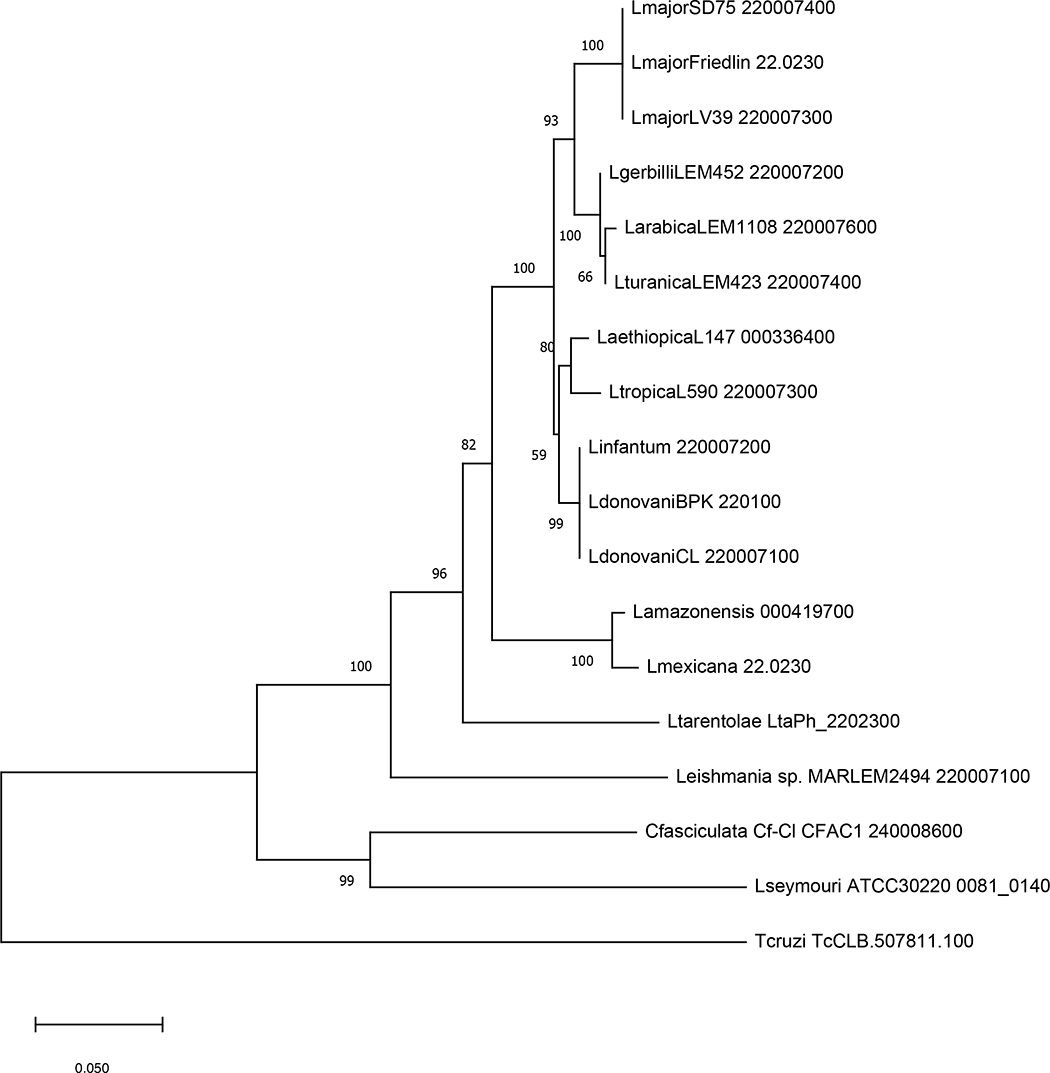

Supplement: S3 Fig — The evolutionary history was inferred using the Neighbor-Joining method. The percentage of replicate trees in which the associated taxa clustered together in the bootstrap test (500 replicates) are shown next to the branches. The tree is drawn to scale, with branch lengths in the same units as those of the evolutionary distances used to infer the phylogenetic tree. The evolutionary distances were computed using the Poisson correction method and are in the units of the number of amino acid substitutions per site. All ambiguous positions were removed for each sequence pair (pairwise deletion option). Orthologs of AAT22 in Leptomonas seymouri, Crithidia fasciculata and Trypanosoma cruzi were used as outgroup. (TIF) [file pntd.0010046.s003.tif]
